# Structure and sequence engineering approaches to improve *in vivo* expression of nucleic acid-delivered antibodies

**DOI:** 10.1016/j.ymthe.2024.11.030

**Published:** 2024-11-19

**Authors:** Michaela Helble, Jacqueline Chu, Kaitlyn Flowers, Abigail R. Trachtman, Alana Huynh, Amber Kim, Nicholas Shupin, Casey E. Hojecki, Ebony N. Gary, Shahlo Solieva, Elizabeth M. Parzych, David B. Weiner, Daniel W. Kulp, Ami Patel

**Affiliations:** 1 Vaccine and Immunotherapy Center, The Wistar Institute, Philadelphia, PA 19104, USA; 2 Department of Cell and Molecular Biology, Perelman School of Medicine, University of Pennsylvania, Philadelphia, PA 19104, USA; 3 Department of Biochemistry and Molecular Biophysics, Perelman School of Medicine, University of Pennsylvania, Philadelphia, PA 19104, USA

**Keywords:** antibody repertoire, antibody, protein engineering, *in vivo* delivery, nucleic acid, DNA, DNA-encoded mAb, gene-delivered biologics, bioinformatics, structure-based design

## Abstract

Monoclonal antibodies are an important class of biologics with over 160 Food and Drug Administration/European Union-approved drugs. A significant bottleneck to global accessibility of recombinant monoclonal antibodies stems from complexities related to their production, storage, and distribution. Recently, gene-encoded approaches such as mRNA, DNA, or viral delivery have gained popularity, but ensuring biologically relevant levels of antibody expression in the host remains a critical issue. Using a synthetic DNA platform, we investigated the role of antibody structure and sequence toward *in vivo* expression. SARS-CoV-2 antibody 2196 was recently engineered as a DNA-encoded monoclonal antibody (DMAb-2196). Utilizing an immunoglobulin heavy and light chain “chain-swap” methodology, we interrogated features of DMAb-2196 that can modulate *in vivo* expression through rational design and structural modeling. Comparing these results to natural variation of antibody sequences resulted in development of an antibody frequency score that aids in the prediction of expression-improving mutations by leveraging antibody repertoire datasets. We demonstrate that a single amino acid mutation identified through this score increases *in vivo* expression up to 2-fold and that combinations of mutations can also enhance expression. This analysis has led to a generalized pipeline that can unlock the potential for *in vivo* delivery of therapeutic antibodies across many indications.

## Graphical abstract

**Figure:**
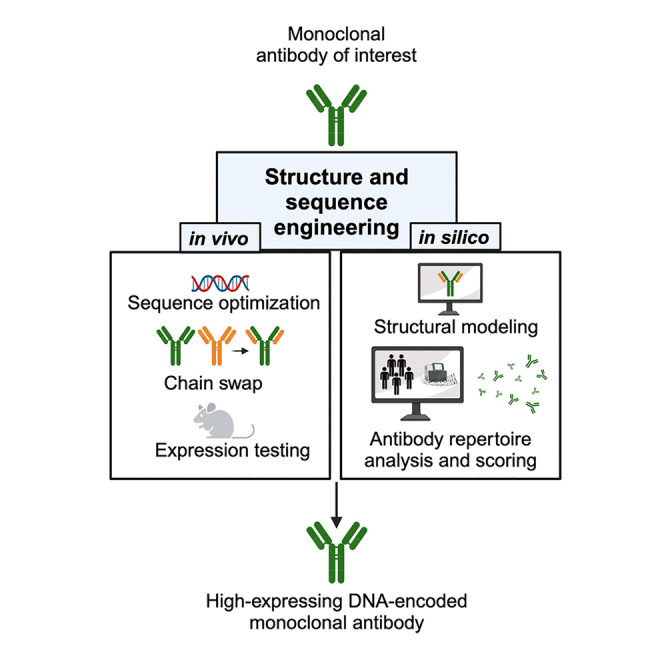

## Teaser

*In vivo* delivery of gene-encoded antibodies can broaden global accessibility to life-saving antibody therapeutics. For gene-encoded platforms, therapeutic efficacy relies on achieving biologically relevant levels of antibody expression. Here, Patel and colleagues develop and employ a predictive pipeline to improve *in vivo* expression with the potential to facilitate clinical translation.

## Introduction

Antibody therapeutics are currently a critical component of care across a variety of fields from cancer to autoimmunity to infectious disease. For cancer, checkpoint blockade antibodies nivolumab, pembrolizumab (anti-PD1), and ipilimumab (anti-CTLA-4) have achieved unprecedented clinical responses and sparked a revolution in cancer treatment^1^^,^^2^^,^^3^^,^^4^^,^^5^; over 55 antibodies in the United States/European Union have been approved for cancer treatment alone.^6^ In autoimmunity, anti-tumor necrosis factor (TNF) antibody adalimumab used in the treatment of ulcerative colitis and Crohn’s disease remains the top-selling monoclonal.^7^^,^^8^^,^^9^ The recent approval of nirsevimab for use in respiratory syncytial virus (RSV) shows the significance of antibodies for infectious disease prevention.^10^^,^^11^ This was also mirrored in the numerous anti-SARS-CoV-2 antibody development efforts initiated during the pandemic.^12^ These examples, and many others, showcase the importance of antibody therapies. They have revolutionized treatment across diverse fields and their continued development and regulatory approval remains critical.

The recombinant monoclonal antibody (mAb) development process includes rigorous antibody biochemical and biophysical property analyses, quality control assessments,^13^^,^^14^ complexities related to dosage and route of administration,^15^^,^^16^ as well as other important manufacturing considerations. Gene-encoded delivery of antibodies *in vivo* may offer a way to circumvent these limitations associated with recombinant antibody production. There are several major platforms that make use of *in vivo* gene-encoded delivery, including viral vectors, mRNA-LNP, and DNA delivery. An antibody of interest is encoded and optimized for direct *in vivo* delivery, where they are expressed ultimately leading to secretion of functional mAb into systemic circulation by host cells.^17^^,^^18^^,^^19^^,^^20^^,^^21^ Gene-encoded mAbs therefore obviate the need for the extensive purification and protein quality control schemes required for recombinant antibodies. Furthermore, gene-encoded antibodies will continue to be secreted for the length of time that the coding sequence remains intact in the host, which changes depending on the delivery platform. Expression is typically on the order of days-weeks (RNA), weeks-months (DNA) or months-years (viral vectors).^19^^,^^22^^,^^23^^,^^24^

Achieving biologically relevant circulating antibody levels is a critical limitation for all *in vivo*, gene-encoded antibody delivery platforms. Akin to any biologic, bioavailability of the drug must be maintained at levels above the minimum effective concentration, or the antibody may not have a therapeutic effect. Therefore, there is a direct link between expression and biologic effect of a therapeutic. Even small improvements to antibody expression, such as 2-fold or greater, could mean the difference between an ineffective therapy (antibody bioavailability below the minimum effective concentration) or an effective therapy (antibody bioavailability above the minimum effective concentration). Currently, it is challenging to predict and manipulate expression levels for *in vivo* delivery platforms, in part because control is lost at the level of dosage (AAV vector genomes, micrograms mRNA or DNA delivered). Understanding this expression limitation is a key barrier to widespread adoption of *in vivo* antibody delivery platforms.

Sequence engineering has long been a strategy to improve protein stability and biophysical characteristics. In the case of recombinant mAbs, this is demonstrated through use of sequence liability analyses to scan for isomerization sites, deamination sites, aggregation propensity, and other biochemical features^25^^,^^26^^,^^27^ in order to improve expression and bioprocess yield. We accordingly evaluated the use of biochemical sequence liability analysis as a predictive feature for *in vivo* antibody expression using the DNA platform. Interestingly, these traditional analyses did not predict features associated with high-expressing antibodies^28^ and there is no observed correlation between *in vitro* and *in vivo* expression. This renders the traditional rules governing antibody expression untenable (Figure S1). We therefore sought alternative structure and sequence engineering pathways to understand what kinds of features might be used to modulate *in vivo* antibody expression for gene-encoded antibody platforms.

**Figure 1:**
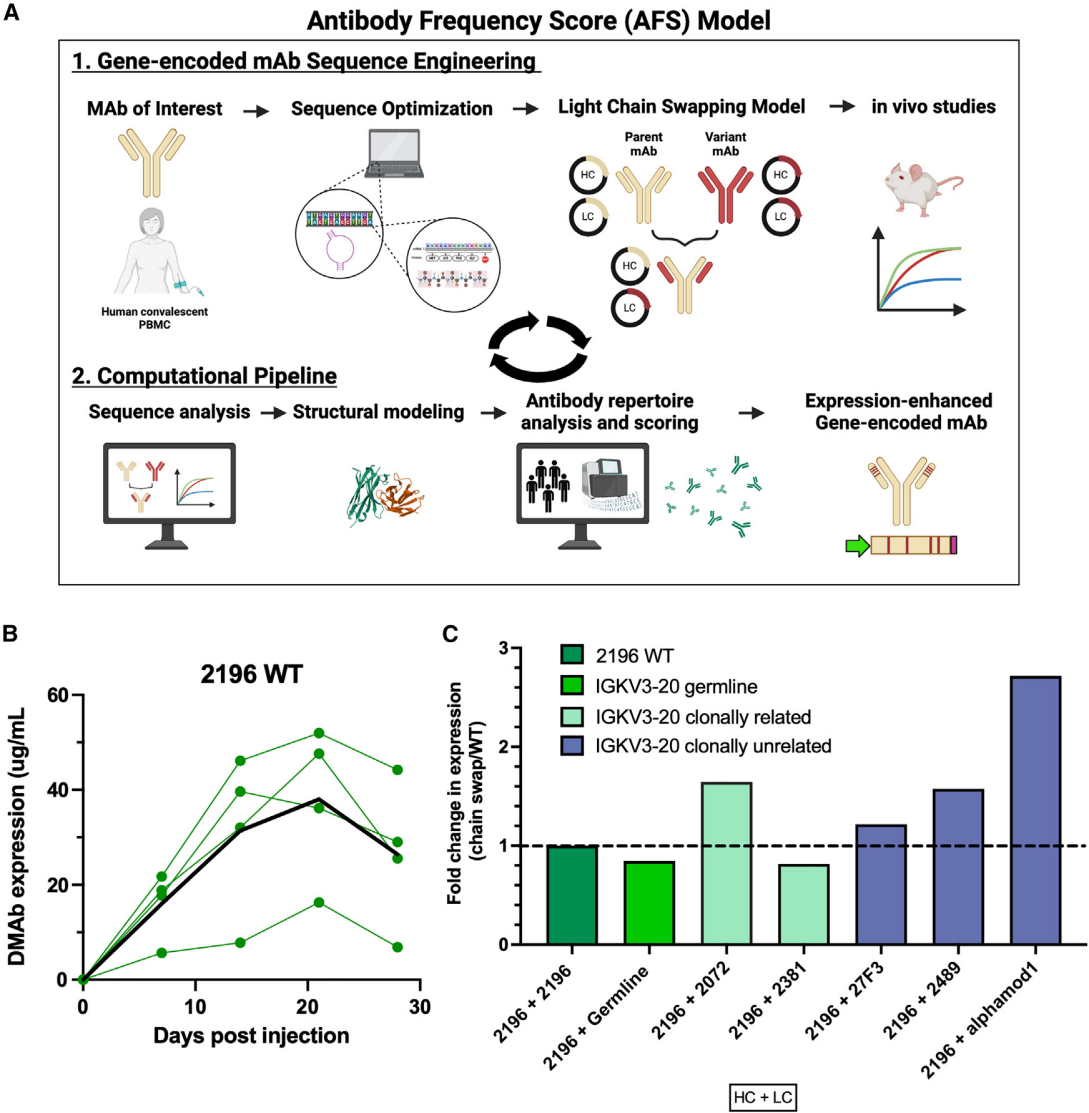
Characterization and analysis of 2196 WT and chain-swapped variants (A) Overview of the pipeline to improve *in vivo* expression. A candidate antibody is first sequence optimized for formulation as a DMAb. Then, chain swap is applied, where HC and LCs of interest are shuffled to identify candidate sequences that improve *in vivo* expression. Sequence analysis and structural modeling of the chain-swapped pairs with improved expression identifies candidate mutations of interest. This guides development of an antibody scoring metric based on millions of naturally occurring antibody sequences. Application of the scoring metric results in selection of new mutations that result in high *in vivo* expression of the candidate DMAb. (B) *In vivo* 2196 WT DMAb expression in 6- to 8-week-old female BALB/c mice. Quantification ELISA was used to assess DMAb serum concentration. Each line is an individual mouse. Bolded line indicates geometric mean. (C) Average fold improvement in expression of *in vivo* LC-swapped variants (*n* = 3 mice/group). LCs were either WT (dark green), germline (bright green clonally related to 2196 (light green), or clonally unrelated to 2196 (purple). Groups are denoted as HC + LC.

Here, utilizing a synthetic DNA delivery platform, we report fundamental studies of antibody structure and sequence repertoires to develop a multi-step pipeline for enhancing antibody expression *in vivo* (Figure 1A). We apply this pipeline to a synthetic plasmid DNA-encoded antibody (DMAb), delivering a well-characterized, public clonotype antibody, COV2-2196^29^^,^^30^^,^^31^; the DNA-encoded version is called DMAb-2196. Following sequence optimization, we take advantage of a unique antibody chain swapping method. This involves selective mismatch pairings of antibody variable heavy (VH) and variable light (VL) chains hypothesizing that native pairings are selected from affinity, but panning with alternative same-germline chains might yield pairings with better expression profiles. From chain swapping, we identified sequence features and motifs that modulated DMAb-2196 expression and used these as a guide to develop an antibody frequency score (AFS) based on analyzing 290 million antibody sequences to identify similar types of mutations. Application of AFS to DMAb-2196 delivery resulted in the identification of both single and combination mutations capable of improving *in vivo* expression up to 2-fold over the wild-type (WT) antibody. In the new and emergent landscape of gene-encoded antibody therapeutics, development of additional tools, such as VH/VL chain swapping and AFS, will be key to enhancing next generation gene-encoded medicines.

## Results

### Immunoglobulin variable chain swapping identifies amino acids that modulate DNA-encoded antibody *in vivo* expression

Previously, we reported that a single amino acid change in immunoglobulin (Ig) framework regions can modulate *in vivo* expression of DNA-encoded antibodies (DMAbs) encoding anti-Ebola virus glycoprotein mAbs from the ZMapp cocktail.^28^ These data further highlighted significant differences in serum expression following *in vivo* delivery of DMAbs encoding Ig from different variable heavy (VH) and variable light (VL) chain families. Building on these initial observations, here we describe development of an antibody VH/VL chain swapping approach designed to specifically elucidate the impact of sequence modifications on *in vivo* gene-encoded antibody expression. We then employ this empiric data to guide development of a computational pipeline (Figure 1A).

DMAb-2196 is a gene-encoded version of mAb COV-2196, the parental mAb to the tixagevimab component of the anti-SARS-CoV-2 Evusheld cocktail (tixagevimab plus cilgavimab).^29^^,^^32^ In combination with DMAb-2130, we previously demonstrated DMAb cocktail protection against SARS-CoV-2 infection in mice and hamsters.^33^ We further demonstrated protection against SARS-CoV-2 associated pneumonia in rhesus macaques.^34^ Multiple public clonotype antibodies have been identified across genetically unrelated human populations, highlighting shared VH/VL gene usage with similar CDR rearrangements.^35^^,^^36^ Guided by this convergent immunity, we focused on the IGHV1-58/IGKV3-20 public clonotype that is frequently represented among anti-SARS-CoV-2 antibodies and selected DMAb-2196 as a gene-encoded model for antibody chain swapping studies.

The *in vivo* pharmacokinetic expression of parent DMAb-2196 is shown in Figure 1B. The antibody chain swapping model leverages natural cognate and mismatch pairing of VH and VL sequences with the goal of identifying sequence changes that modulate *in vivo* DMAb expression levels. In these studies, we focused on the impact of DMAb light chain (DMAb-LC) variants on *in vivo* expression. Synthetic DMAb-LC variants were engineered to encode selected IGKV3-20 genes that (1) arise from the same B cell clone as parent mAb COV-2196 (clonally related) and target the SARS-CoV-2 receptor binding domain, (2) clonally unrelated mAbs from the same dataset that target the SARS-COV-2 N-terminal domain,^30^^,^^31^ (3) clonally unrelated mAbs targeting other pathogens (influenza A virus hemagglutinin protein and *Staphylococcus aureus* alpha toxin),^37^^,^^38^ as well as (4) the unrecombined germline IGKV3-20 sequence (Figure S2A). To minimize overall sequence variation due to codon bias, DMAb-2196-LC base nucleotides were used as a template and the most frequent human codons were selected for variant DMAb-LC-IGKV3-20 amino acids. Next, the DMAb heavy chain (DMAb-HC) of 2196 was combined with matched DMAb-2916-LC or mismatched variant DMAb-LCs. A comparison of the mutations relative to 2196 can be found in Figure S2B.

Enhanced *in vivo* expression was observed with several DMAb-2196-HC plus variant DMAb-LCs combinations (Figure 1C). DMAb-2196-HC in combination with clonally related DMAb-2072-LC resulted in 1.34-fold *in vivo* expression improvement over WT DMAb-2196-HC+LC. All clonally unrelated pairings showed increases *in vivo* expression; DMAb-2196 HC + DMAb-alphamod1-LC demonstrated the greatest magnitude increase with a 2.72-fold improvement over WT matched pairing. Notably, this deliberate mispairing method through antibody chain swapping enables identification of amino acids that contribute to *in vivo* expression. *In vivo* expression was modulated with 4/6 DMAb-LC variants compared with the WT construct. This chain swapping method can therefore be applied to study other antibody VH and VL pairings to further dissect sequence properties that contribute to gene-encoded antibody expression.

### CDRL1-and CDRL3-focused rational design

**Figure 2:**
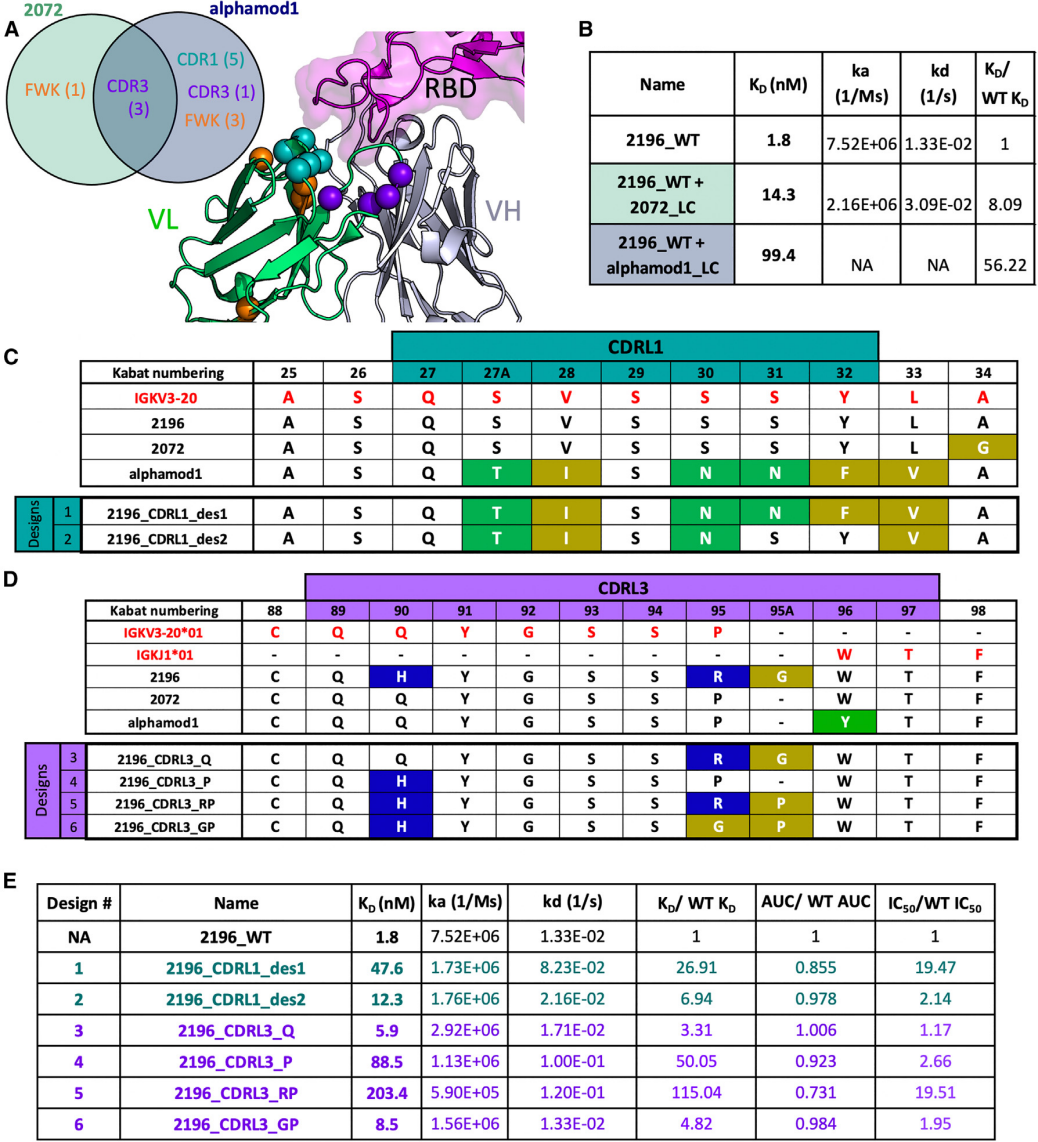
CDRL1-and CDRL3-focused rational design (A) Mutations from 2072 and alphamod1 LCs mapped onto the crystal structure of 2196 (PDB: 7L7E). Mutations are shown as spheres. The RBD of SARS-CoV-2 is shown as a surface-filled model. The Venn diagram inset shows the region and number of mutations contributed by either 2072, alphamod1 or both LCs. (B) Binding kinetics summary of chain-swapped variants. K_D_ of designed variants were divided by K_D_ of 2196 WT to determine ratio. (C) Sequence overview of new CDRL1-focused variants. Germline is shown in red. Any mutations from germline are colored by polarity. Positions of CDRL1 residues are colored in teal. Kabat numbering is used. (D) Sequence overview of new CDRL3-focused variants. Colors are as in (A) and positions of CDRL3 residues are colored light purple. Kabat numbering is used. (E) Binding kinetics summary of designed variants. K_D_ of designed variants were divided by K_D_ of 2196 WT to determine K_D_ ratio. ELISAs were run to calculate AUC and were divided by 2196 WT AUC to determine AUC ratio. ELISA samples were run in duplicate.

The LCs of the highest-expressing chain-swapped variants were analyzed. Relative to DMAb-2196-LC, most differences present in DMAb-2072-LC and DMAb-alphamod1-LC were in the CDR loops, with only a handful of mutations present in the framework regions. Mutations common between the two LCs were present exclusively in the CDRL3 loop (R95P, 95AΔ, Kabat numbering) (Figure 2A). While our analysis revealed common amino acids associated with increased *in vivo* expression, there was a decrease in antigen binding affinity associated with amino acid substitutions or deletions in the chain-swapped variants: WT 2196 K_D_ was 1.8 nM, with K_D_s for 2196 HC + 2072 LC and 2196 HC + alphmod1 LC measured at 14.3 nM and 99.4 nM, respectively (Figures 2B and S3). The shortened CDRL3 loop length relative to the WT antibody might partially explain the reduction in binding affinity observed for both chain-swapped variants. Indeed, structural modeling of these CDRL3 loops with AlphaFold^39^^,^^40^ is consistent with this hypothesis. Similar predictions were also obtained using IgFold^41^ (data not shown). For both algorithms, the modeled structure reveals that residues in the CDRL3 loop do not directly contact antigen (receptor binding domain [RBD] of SARS-CoV-2). However, the longer loop length of DMAb-2196-LC appears to “hug” the RBD and create a binding groove that may not exist within DMAb-2072-LC or DMAb-alphamod1-LC’s shorter CDRL3 loops (Figure S4). The DMAb-alphamod1-LC variant has an additional loss in binding affinity that is likely attributable to unique CDRL1 mutations, as two of these residues, S31 and Y32, directly contact RBD.^29^ There are also two mutations in Vernier zones, regions directly adjacent to CDRs that can affect CDR loop conformation and therefore antigen binding.^42^

Given these observations, we designed minimal sets of mutations into DMAb-2196 LC that could recover binding affinity while also preserving the *in vivo* expression enhancement identified by VH/VL chain swapping. As the majority of mutations in the DMAb-LC variants were present in the CDRL1 and CDRL3 loops, we chose to focus our rational design efforts in those locations. We mutated the CDRL1 of 2196 with alphamod1’s CDRL1 and CDRL1-adjacent residues to create new variant 2196_CDRL1_des1 (hereafter “Des1”). We furthermore created a partially reverted design variant 2196_CDRL1_des2 (hereafter “Des2”), which reverts residues 31 and 32 back to 2196 WT amino acid identities as they form contacts with the RBD and may contribute to binding affinity^29^ (Figures 2C and S5). To design new CDRL3 variants, we analyzed the structure of the CDRL3 loop and observed that most mutations were present at the base of the CDRL3 (Figure 2A). We created a variant of DMAb-2196-LC with an H90Q mutation, as glutamine is present at this position in DMAb-2072-LC and DMAb-alphamod1-LC. This variant became 2196_CDRL3_Q (hereafter “Q”). Of note, this mutation represents a reversion to the IGKV3-20 germline residue (Figure 2D). We also created three additional designs based on positions 95 and 95A. One variant of 2196 recapitulated the CDRL3 from 2072 and alphamod1 by shortening the CDRL3 loop length and introducing a proline at residue 95 to create new variant DMAb-2196_CDRL3_P (hereafter “P”). However, we hypothesized that the shorter loop length might remove the ability of the CDRL3 to “hug” RBD (Figure S4) and thus result in a loss of binding affinity. To address this potential issue, we used structural modeling to create two additional variants that maintained the proline, but also restored the longer loop length present in 2196 WT (Figure S6). One variant contains the arginine from 2196 at position 95 (2196_CDRL3_RP) and one with a glycine residue at position 95 (2196_CDRL3_GP) (hereafter “RP” and “GP,” respectively) (Figure 2D).

We first assessed binding affinity to the RBD of all new antibody variants through both ELISA and SPR. These assays were used as a filtering step to determine which constructs to test for *in vivo* expression (Figures 2E, S7, and S8). The Q, GP, and Des2 variants bound RBD similarly to WT 2196 according to the area under the curve (AUC) (AUC ratio >0.975) and half maximal inhibitory concentration (IC_50_) (<2.14-fold) analysis with K_D_s of 5.9, 8.5, and 12.3, respectively. These values were expected, as Q had a mutation that was not involved in RBD binding, GP maintains the longer loop length hypothesized to help hug the RBD, and Des2 retains WT residues shown to be directly involved in RBD binding. The rest of the variants show weaker binding affinity, ranging from a 27- to 115-fold decrease in binding affinity (Figures 2E and S8). Neutralization of SARS-CoV-2 pseudovirus was also assessed for WT, Q, GP, and Des2 with IC_50_s of 0.08, 0.09, 1.12, and 0.49, respectively (Figure S9A).

### *In vivo* expression of rationally designed variants

**Figure 3:**
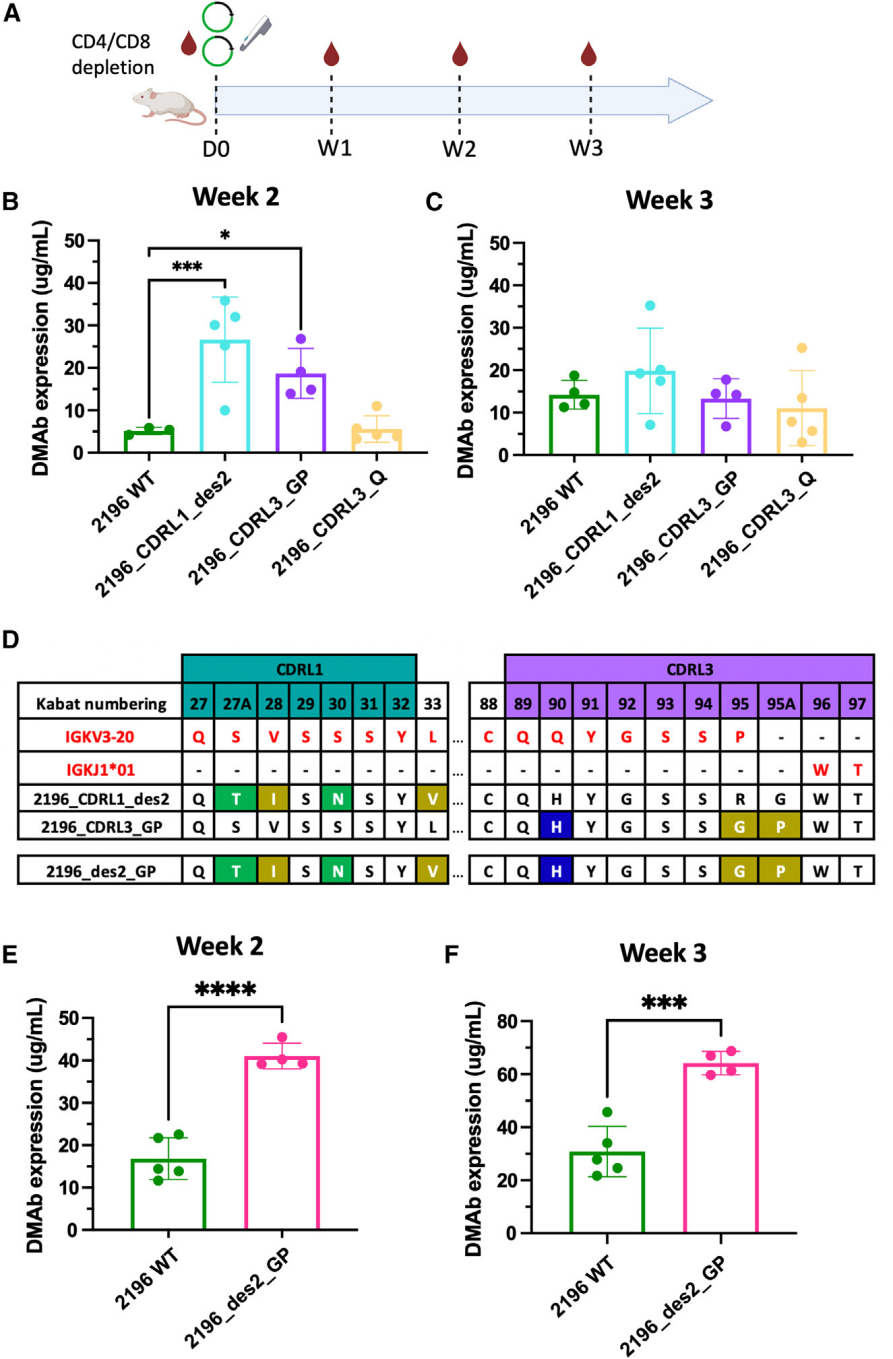
*In vivo* expression of rationally designed variants (A) Bleed schedule for testing *in vivo* expression of CDRL1 and CDRL3 rationally designed variants. On day 0, mice were pre-bled and received CD4+/CD8+ T cell depletion antibodies, then were injected with the relevant DMAb plasmids, followed by electroporation. Mice were then bled weekly as indicated. *In vivo* DMAb expression of CDRL1 or CDRL3 rationally designed variants at (B) week 2 or (C) week 3 in 6- to 8-week-old female BALB/c mice (*n* = 5 mice/group). Mean and standard deviation shown. Quantification ELISA was used to assess DMAb serum concentration. Mice that did not respond fully to depletion were excluded from analysis. One-way ANOVA with post hoc analyses was conducted. ∗*p* < 0.05, ∗∗∗*p* < 0.001. (D) Sequence overview of new combined variant 2196_des2_GP. Germline is shown in red. Any mutations from 2196 WT are colored by polarity. Positions of CDRL1 residues are colored in teal; CDRL3 in purple. Kabat numbering is used. *In vivo* DMAb expression of 2196_des2_GP at (E) week 2 or (F) week 3 in 6- to 8-week-old female BALB/c mice (*n* = 5 mice/group). Mean and standard deviation shown. Quantification ELISA was used to assess DMAb serum concentration. Mice that did not respond fully to depletion were excluded from analysis. Two-tailed t tests were conducted. ∗∗∗*p* < 0.001, ∗∗∗∗*p* < 0.0001.

We selected the three variants (Q, GP, Des2) with similar K_D_ and AUC values to 2196 WT to study the impact on *in vivo* expression and formulated these as DMAbs. Mice were injected with the DMAb-HC/LC plasmids of interest and monitored weekly for presence of human IgG in sera as in Figure 3A. There was very little difference in IgG expression between DMAb-Q and DMAb-2196-WT. In contrast, both DMAb-Des2 and DMAb-GP showed significant improvements in expression over time. DMAb-Des2 had the overall highest improvement in expression over WT, reaching average peak expression levels of 26.6 μg/mL (Figure 3B). This supports the concept that the additional CDRL1 mutations present in alphamod1 were at least partly responsible for the higher expression observed with the DMAb-2196 HC + DMAb-alphamod1 LC combination. We additionally identified residues in the CDRL3 that contributed to improved expression through variant DMAb-GP, which averaged 18.7 μg/mL of IgG expression at peak expression.

While these differences in expression start to decline by week 3 (Figure 3C), this may be due to incomplete CD4+/CD8+ T cell depletion, resulting in the development of mouse anti-human antibodies and the subsequent clearance of IgG.^43^ In a species-matched model, it is likely that higher levels of IgG expression will persist, as development of anti-drug antibodies (ADAs) is less likely to occur.^44^^,^^45^^,^^46^ When considering clinical translation, there is minimal concern for ADAs in humans, as each DMAb variant in this study is derived from fully human antibody sequences.

Given the significant expression increase observed from CDRL1-focused design (DMAb-Des2) and CDRL3-focused design (DMAb-GP), we combined both these sets of mutations into a single new variant DMAb-2196_des2_GP (hereafter “Des2_GP”) (Figure 3D) to understand if expression-enhancing mutations could be additive. The recombinant mAb was produced and purified to assess binding affinity; this was similar to the K_D_s of individual variants Des2 and GP (Figure S10). Following an identical experimental design to Figure 3A, DMAb-Des2_GP was evaluated *in vivo*. The expression enhancements from this combined variant were significant, resulting in an average of 41.1 μg/mL of IgG expression at week 2 (Figure 3E), and, interestingly, a peak at week 3 with expression levels of 64.2 μg/mL (Figure 3F). DMAb-Des2_GP shows around a 2-fold increase over WT DMAb-2196 expression levels. However, because both Des2 and GP individual variants had reduced binding affinity and neutralization compared with WT 2196, the combined variant Des2_GP accordingly also had a binding affinity and neutralization reduction (Figures S9A and S10).

### Amino acid frequency analysis of 2196-like IGKV3-20 antibodies

Chain swapping followed by sequence analysis and structural modeling yielded interesting mutations that significantly improved *in vivo* expression over WT DMAb-2196. As alphamod1 and 2072 both have IGKV3-20 LCs, we reasoned that other IGKV3-20 LCs might also contain expression-enhancing mutations. We therefore surveyed millions of IGKV3-20 antibody sequences with a goal of identifying new mutations that improve *in vivo* antibody expression with no impact on binding affinity. By surveying a large dataset of antibody sequences that target a diverse array of antigens, we hypothesized that any commonly observed mutations should not impact binding capability of antibodies harboring those mutations. We sought to develop a scoring metric to help find mutations of interest by identifying positions with increased mutational frequencies. We further utilized previously discovered expression-improving mutations in DMAb-Des2-GP as a benchmark and guide in development of this scoring metric.

**Figure 4:**
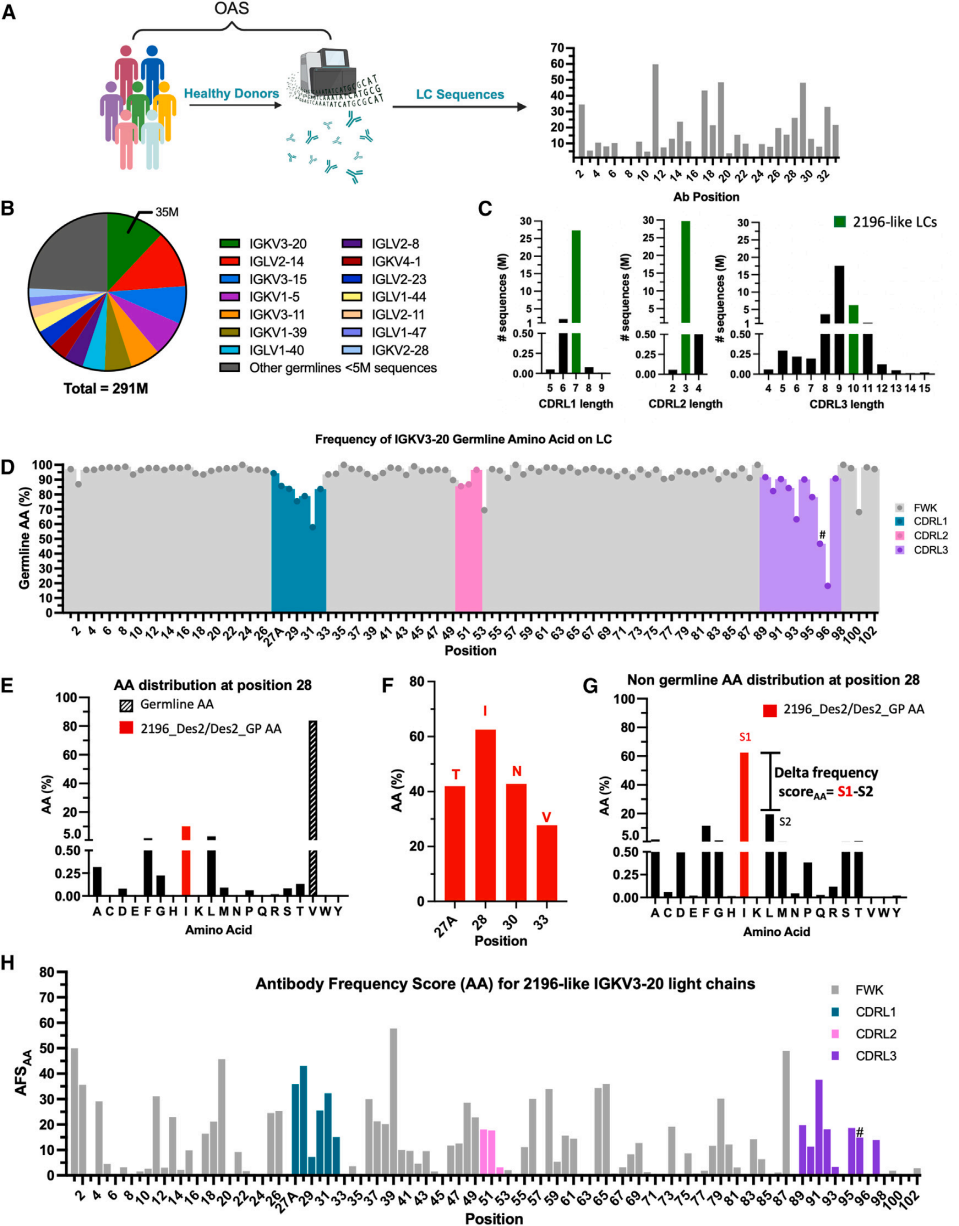
Amino acid frequency analysis of 2196-like IGKV3-20 antibodies (A) Overview of frequency alignment process. The observed antibody space (OAS) database collates data from studies sequencing antibody repertoires. After sorting by healthy individuals, the database has sequences from 291 million unpaired LCs. We wrote a script that is capable of searching through these sequences by light chain V-gene, J gene, or sequence motif and then tabulating the frequency of each amino acid at every position in the antibody. This metric is used to develop a frequency score (details below). (B) LC germlines in OAS database. The healthy LC OAS sequences were searched by antibody germline. The 14 germlines that have over 5 million sequences in the database are shown in color; all remaining antibody germlines with less than 5 million sequences are colored in dark gray. (C) Distribution of IGKV3-20 antibody CDR lengths. The IGKV3-20 subset of light chains was searched for motifs corresponding to CDRL1, 2, or 3 regions and by specific CDR length. The CDR lengths possessed by 2196 are colored green. (D) Frequency of germline amino acid for 2196-like IGKV3-20 LCs by position. Following the workflow in (A), the frequency of germline amino acid at each position was calculated for IGKV3-20 and represented as a percentage of searched sequence hits. Positions are colored by CDR or FWK. The J gene considered as germline was matched to 2196-like antibodies (J1). #VJ junction. Proline was considered germline here (resulting from direct VJ gene joining with no insertions or deletions). (E) Amino acid frequency distribution at position 28. The frequency of every amino acid at a particular position was analyzed as in (A). Germline amino acid (V) was most frequent, followed by I, found in high-expressing construct 2196_Des2 and Des2_GP. (F) Non-germline frequency of CDRL1 mutations of interest. The amino acid identity for mutations in the CDRL1 region of high-expressing Des2 and Des2_GP variants is shown in red, per position. Frequency of each amino acid is expressed as a percentage of sequences that do not contain the germline amino acid at the identified position. (G) Non-germline amino acid frequency distribution at position 28. Frequency of each amino acid is expressed as a percentage of sequences that do not contain the germline amino acid. The most frequent non-germline amino acid (I) was termed S1 and the second most frequent termed (L) S2. The difference in S1-S2 became a delta frequency store, indicative of potential preference for a given non-germline amino acid. (H) AFS_AA_ for 2196-like IGKV3-20 LCs by position. A scaling factor was introduced to account for false positives in the delta frequency score that could be due to having more codons encode for the same amino acid, suggesting preference for a particular amino acid was due to chance rather than selection. Any position where germline was not the most frequent amino acid was omitted from analysis. #VJ junction.

To facilitate our analysis of IGKV3-20 antibody sequences with diverse antigenic targets, we utilized the Observed Antibody Space (OAS) database,^47^^,^^48^ which is one of the largest antibody sequence databases with extensive annotations for ease of sequence extraction. The sequencing data was composed of LC sequences that had been filtered to only include healthy donors, which avoids introducing potential sequence bias associated with a particular disease state (Figure 4A). We identified an initial pool of 291 million unpaired LC sequences for analysis. We next wanted to determine how many naturally occurring antibody sequences shared 2196’s immunogenetics to ensure sufficient sequencing data for further downstream analysis. The germline of all 291M light chain sequences was determined (see materials and methods). Fourteen antibody V-gene families comprise more than 75% of LC sequences, with IGKV3-20 as the most common antibody germline (35M sequences) (Figure 4B). This aligns with other repertoire studies showing frequent usage of IGKV-3 family genes in human repertoires.^49^^,^^50^

We further assessed the distribution of IGKV3-20 CDRL1, 2, and 3 lengths as compared with 2196. We observed that 2196 has the most common CDRL1 and CDRL2 length and the second most common CDRL3 length (Figure 4C). It is not altogether surprising that a CDRL3 of length 9 is the most common as this is the germline-encoded length,^51^ representing a direct in-frame VJ sequence joining. To discover patterns useful for applying to 2196, we decided to restrict our data to antibodies that contained identical CDRL lengths to 2196 (IGKV3-20 antibody with CDRL1 length 7, CDRL2 length 3, and CDRL3 length 10, hereafter referred to as “2196-like” sequences). This resulted in close to 6 million 2196-like sequences for analysis.

The frequency of every amino acid at every position in the VL was then determined. Across all 2196-like, IGKV3-20 antibodies, germline amino acids are observed at extremely high frequencies in the framework region, with a lower degree of germline amino acids observed in the CDR regions (Figure 4D), as expected. We next assessed the frequency distribution of amino acids at positions that are mutated in our high-expressing DMAb-2196_Des2 and DMAb-2196_Des2_GP variants. An example of this is shown for position 28 (Figure 4E), where we made the striking observation that isoleucine, the amino acid found in DMAb-2196_Des2/Des2_GP is the *most frequent non-germline-encoded amino acid.* In other words, this mutation from germline is very commonly found in IGKV3-20 antibodies (*n* = 581,654). Indeed, for antibody sequences that contain a mutation at position 28, an isoleucine is found *>60% of the time* (Figure 4F). We conducted similar analyses for positions containing the other DMAb-2196_Des2/Des2_GP mutations and observed that these mutations from germline are also found at high rates in naturally occurring antibody sequences (Figure 4F). This suggests a preference for these particular mutations, as they are found in such a high proportion of 2196-like antibodies which diverse antigenic targets.

We reasoned that a strong selection for a mutation could be identified by comparing the degree of preference for that amino acid over others. We therefore analyzed the 2196-like sequences to look for differences in frequencies; an example of this analysis at position 28 is shown in Figure 4G. We created a delta frequency score, indicative of preference for a given mutation, which is calculated as the difference between the most frequent non-germline amino acid, S1, and the second most frequent non-germline amino acid, S2.

However, we were concerned that there might be artificial false positives in this frequency score due to redundancy in the codon table and ease of achieving a given amino acid mutation from germline, rather than actual selection for the amino acid. To avoid these types of false positives, we normalized our delta frequency score in proportion to how many codons encoded for a given amino acid (see materials and methods, example at position 89 in Figure S11). The results of the adjusted score, taking codon bias into account, was termed amino acid-based antibody frequency score (AFS_AA_) (Figure 4H).

The AFS_AA_ score identifies several positions with a strong preference for a particular non-germline amino acid; these preferences are most prevalent at several positions in the framework region, as well as in the CDRL1 region (Figure 4H).This is particularly notable because these sequence comparisons are cross-donor; a total of 59 out of 70 possible donors contributed to the sequences analyzed (Figure S12). Since CDRs can engage antigen and are often the most variable part of the antibody, it is remarkable that there is any preference at all for amino acids in this region across donors. This enrichment is suggestive of the idea that there may be some degree of non-affinity-based selection in naturally occurring antibodies. The enriched positions do not correspond to allelic differences (Figure S13), further lending credence to the idea of selection.

### Development and application of AFS_NT_ to improve *in vivo* expression

**Figure 5:**
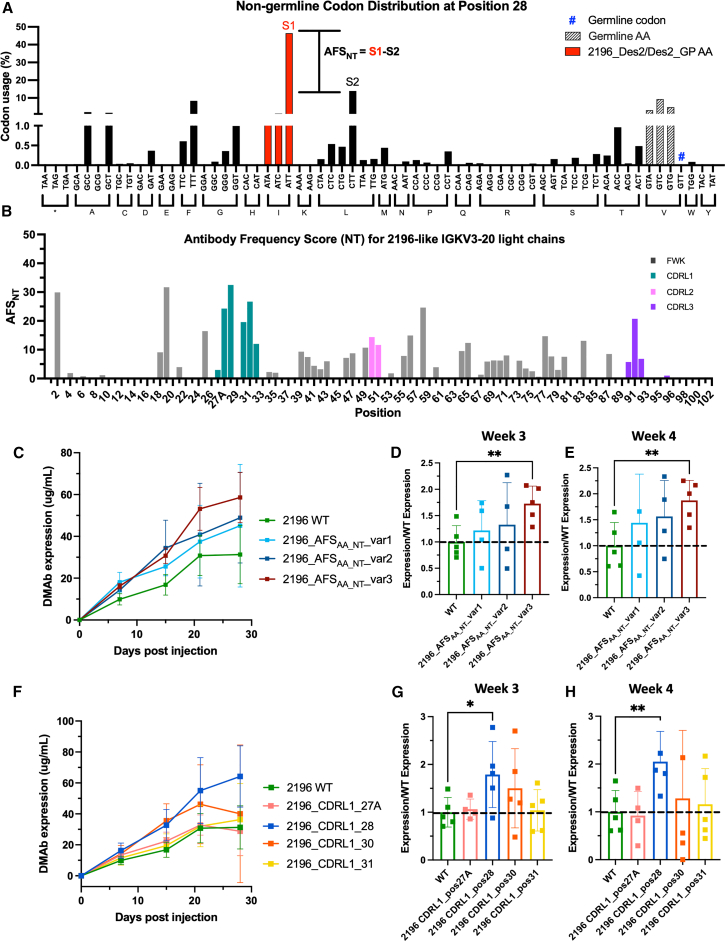
Development and application of AFS_NT_ to improve *in vivo* expression (A) AFS_NT_ development. As in 4E, the frequency of every codon at a particular position (here, position 28) was analyzed and then expressed as % non-germline codon. The most frequent non-germline codon (ATT) was termed S1 and the second most frequent (CTT) termed S2. The difference in S1-S2 became AFS_NT_, a frequency score derived from codon usage. #Germline codon. (B) AFS_NT_ for 2196-like IGKV3-20 LCs by position. If S1 was a codon that still encoded the germline amino acid, this position was excluded from analysis as it indicates germline is preferred. (C) *In vivo* expression of 2196 variants derived from AFS_AA_ and AFS_NT_ scores in 6- to 8-week-old female BALB/c mice (*n* = 5 mice/group). Quantification ELISA was used to assess DMAb serum concentration. Group mean and standard deviations shown. Any mouse not responding to depletion antibodies was excluded from analysis. Fold improvement in expression over WT for AFS_AA_ and AFS_NT_ variants at (D) week 3 or (E) week 4. (Expression)/(WT average expression) was calculated for each mouse. Mean and standard deviation shown. Any mouse not responding to depletion antibodies was excluded from analysis. One-tailed t tests were conducted. ∗∗*p* < 0.01. (F) *In vivo* expression of single mutation CDRL1 variants in 6- to 8-week-old female BALB/c mice (*n* = 5 mice/group). Quantification ELISA was used to assess DMAb serum concentration. Group mean and standard deviations shown. Any mouse not responding to depletion antibodies was excluded from analysis. Fold improvement in expression over WT for CDRL1 single position mutants at (G) week 3 or (H) week 4. (Expression)/(WT average expression) was calculated for each mouse. Mean and standard deviation shown. Any mouse not responding to depletion antibodies was excluded from analysis. One-tailed t tests were conducted. ∗*p* < 0.05, ∗∗*p* < 0.01.

Given the complicating factors of codon bias in AFS_AA_ analysis, we decided to perform a secondary analysis using nucleotide sequences directly. Analogous to the development of AFS_AA_, we determined the distribution of non-germline codons at all positions. Strikingly, we observe in position 28 that >*40%* of 2196-like antibody sequences utilize one codon (ATT) over any other (Figure 5A). While the germline codon at position 28 in IGKV3-20 antibodies is “GTT,” which codes for valine, the most frequent mutated codon is “ATT,” which encodes for isoleucine (>40%) and requires only a single base change. For a single base change from the germline codon to occur without selection, it would be expected at a frequency of 11% (1 of 9). The other eight single base change codons are observed at frequencies from 0.35% to 9.4%, including the three other codons that encode for the germline amino acid valine. Thus, we sought to identify positions with perceived selection based on codon frequency differences. We next developed a new score based on the difference between the most frequent non-germline codon (S1) and the second most frequent non-germline codon (S2). This difference represents preference not only for a specific amino acid, but for a specific codon at a given position, and is referred to as a nucleotide-based antibody frequency score (AFS_NT_) (Figure 5B).

Analysis of AFS_NT_ provided additional insight into selection of mutations over germline. We observed that in some cases high frequency codon mutations are silent, encoding for the germline amino acid. This effect can be observed at positions 39, 87, and 89 (Figure S14). These cases provide support for the idea that the germline amino acid can be preferred at that position, information that is not captured through AFS_AA_. The additional granularity of AFS_NT_ demonstrates that these positions are less strongly selecting for the mutation highlighted in AFS_AA_ (Figures 4H and 5B). However, analysis of AFS_NT_ at all positions demonstrates that there are a handful of intriguing framework positions (e.g., 2, 19, 58) that show clear enrichments and high scores. The Y to F mutation at position 91 in the CDRL3 also has a high AFS_NT_ score; however, such a mutation may impact antigen binding. Interestingly, the CDRL1 contains positions with the highest enrichment scores with position 28 being the overall highest AFS_NT_ score (Figure 5B). Taken together, this supports the idea that both AFS_AA_ and AFS_NT_ could be used to identify positions of interest for mutations that would lead to higher expressing variants.

To examine our overarching hypothesis that frequent cross-donor, antigen-agnostic mutations may enhance expression, we used both AFS_AA_ and AFS_NT_ to identify positions in the 2196 VL that had high enrichment scores to test as new variant DMAbs. Positions with high AFS_AA_/AFS_NT_ scores that had not been mutated in prior variants were mapped to the structure of 2196 to assess possible impacts on binding affinity or antibody framework fidelity. We then sought to investigate these mutations further as there were no obvious potential structural perturbations from our modeling (Figure S15). We designed variant DMAb-2196_AFS_AA_NT__var1 from high AFS_AA_/AFS_NT_ scores that focus on CDRL1 mutations (positions 27A,28 and 31). A second variant, DMAb-2196_AFS_AA_NT__var2, builds on mutations from high-expressing variant DMAb-Des2, but further incorporates the framework mutations at positions 2 (high in both AFS_AA_ and AFS_NT_), 39, and 56 (high in AFS_AA_). Finally, DMAb-2196_AFS_AA_NT__var3 includes the mutation at position 2, as well as framework positions 49, 76, and 83 (high in AFS_AA_). Position 76 was identified using slightly altered OAS V-gene search criteria to handle variability in antibody sequencing methods (see materials and methods, Figure S16).

Next, we sought to examine our designs as DMAbs to assess the impact on *in vivo* antibody expression. We followed antibody expression out to week 4 to confirm expression differences between variants derived from AFS predictions. The designed variants demonstrate improved expression over WT, with peak expression occurring around week 3 and week 4 (Figure 5C). Peak expression ranges from 45 to 58 μg/mL for all new AFS_AA_NT_ variants. Fold over WT DMAb-2196 expression was calculated for each of these variants for week 3 (Figure 5D) and week 4 (Figure 5E). The design DMAb-2196_AFS_AA_NT__var3 showed significant improved expression over WT for both timepoints, with a 1.9-fold improvement in expression. Importantly, and in contrast to earlier chain-swap and rational design antibodies, the AFS-variant antibodies show equivalent binding and neutralization to WT (KDs of 1.4, 1.6, and 3.2 nM compared with the WT KD of 1.8) (Figure S17A); (IC_50_ of 0.08 ng/mL, 0.05 ng/mL, and 0.06 ng/mL compared with WT IC_50_ 0.08 ng/mL) (Figure S9B).

To assess the enrichment-expression link more directly, we sought to deconvolute effects from multiple mutations by generating single mutation variants of DMAb-2196. Further, though we did not see a decrease in binding affinity with our new combination variants, we thought it important to identify the most minimal set of mutations that modulate expression. Given that both AFS_AA_ and AFS_NT_ show a large degree of both enrichment and agreement at positions in the CDRL1, we decided to test each position as a standalone mutation. Positions 27 and 29 in the CDRL1 are not enriched, so these were excluded from this analysis. Each single mutant was assessed for *in vivo* expression in the DMAb format. Interestingly, single mutations to DMAb-2196 positions 28 and 30 show improved expression, with significantly higher peak expression of 64.2 μg/mL for position 28 (Figure 5F). Single mutations at positions 27A and 31 show nearly identical expression to WT DMAb-2196. Peak expression for all variants occurs around week 3 or week 4. Fold over DMAb-2196-WT expression was calculated for each of these variants for week 3 (Figure 5G) and week 4 (Figure 5H), with significant improvement in expression for position 28. We have therefore identified a *single position* that is capable of improving *in vivo* expression 2.1-fold over WT using AFS_AA_NT._ We observed no decrease to RBD binding or neutralization for most of these positions, with position 30 having a slight reduction and position 27A having a 10-fold improvement in neutralization potency (Figures S9B and S17B).

Overall, structural and rational design of the 2196 LC allowed for identification of variants with significantly improved expression, though with some reduction in binding affinity to WT RBD. We were able to extract important mutations of interest as a guide and compare these mutational signatures to selection of non-germline amino acids in naturally occurring antibodies. This ultimately led to the development of two scoring functions (AFS_AA_ and AFS_NT_). Application of these scores has allowed us to generate variants of DMAb-2196 with improved *in vivo* expression, yet no loss to binding affinity or neutralization. This approach is promising for optimization of *in vivo* delivery of IGKV3-20 DMAbs. Further, AFS_AA_/AA_NT_ scores can be generated for heavy chains to potentially allow further increases in expression and thus be applied to improve *in vivo* delivery of many other antibody families targeting a broad range of antigens.

## Discussion

Here, we have developed and applied two methods to improve the *in vivo* expression of gene-encoded DMAbs, using anti-SARS-CoV-2 DMAb-2196 as a proof of concept. Light chain swapping of clonally related and unrelated antibody chains resulted in a maximal expression enhancement of 2.72-fold over the WT antibody, though with a concomitant reduction in binding affinity to WT RBD (Figures 1C and 2B). Sequence analysis and structural modeling of mutations from these chain-swapped LCs resulted in selection of a pared down set of amino acids, yielding new variants with significant improvements to *in vivo* expression (Figures 3B, 3C, 3E, and 3F). Finally, using these key discovered mutations as a guide, we developed antibody scoring metrics AFS_AA_ and AFS_NT_, to assess the predilection of IGKV3-20-germline antibodies to mutate from germline at positions across the entire V-gene (Figures 4G, 4H, 5A, and 5B). Application of these metrics to DMAb-2196 resulted in a single mutation conferring a 2.1-fold improvement in expression over WT, with no effect on binding affinity or neutralization (Figures 5F, 5G, S9B, and S17B). While our pipeline has four main steps: standard DMAb sequence optimization light chain swapping, sequence and structure engineering, and AFS_AA/NT_, these methods can also be used independently to improve *in vivo* antibody expression.

We have observed that antibodies with the same germline heavy and/or light chains can have strikingly different *in vivo* expression profiles. Swapping chains of genetically related antibodies is an efficient way of identifying mutations transferable between antibodies. By creating multiple synthetic DMAb-LCs for 2196, we found many with higher expression. This allowed us to discern shared mutations responsible for improved *in vivo* expression. A second insight is that coupling chain swapping with structural modeling can aid identification of mutations that may impact binding. Last, chain swaps are likely generalizable such that mutations found in chain swaps of 2196 (containing a IGKV3-20 light chain) can be re-used for improving expression of other IGKV3-20 containing antibodies.

The AFS method described here is also not limited to improving 2196-like antibodies. The first augmentation for AFS_AA_ and AFS_NT_ is the allowance of IGKV3-20 antibodies of *any* CDRL3 length, not just matched to 2196 (length 10 CDRL3), which allows for higher coverage of positions of interest. To address the potential for generalizability of AFS, we have also extended our analysis to the most common antibody germlines present in the OAS (IGLV2-14, IGKV3-15, IGKV1-5, IGKV3-11, IGKV1-39, IGLV1-40), which, together with IGKV3-20, cover over 50% of light chain sequences (Figures 4B, S18, and S19). These analyses were also conducted to be independent of CDRL3 length. In addition to LC V-gene analysis, it will be important to extend the AFS pipeline to obtain information derived from mutations found in HC germline V-genes. It will be interesting to assess if combining LC and HC AFS-derived mutations synergize and provide even higher *in vivo* expression profiles of DMAbs.

This study raises interesting mechanistic questions on *in vivo* expression improvements. For example, how are sequence features from naturally occurring antibodies linked to antibody expression? One possibility might be through some degree of B cell receptor (BCR) selection. BCR clustering and accumulation of productive somatic mutations are signals for positive selection and survival of germinal center B cells.^52^^,^^53^^,^^54^^,^^55^ Deleterious mutations can also be acquired, leading to negative changes in BCR structure that result in malformed, truncated, or non-expressed light chains, ultimately leading to B cell apoptosis.^56^^,^^57^ Therefore, mutations that affect BCR expression levels may modulate levels of antigen acquisition and proliferation signals and persist in sequencing data that is ultimately captured by AFS_AA/NT_. A second mechanistic angle could be in specific codon usage. The overall codon optimization of a sequence can affect expression due to local changes in tRNA concentration that might initiate translational pausing and affect overall translation rate.^58^^,^^59^ Further, recent studies link *in vivo* antibody expression to codon dependance,^60^ and to codon optimality.^61^ Interestingly, our codon optimization of 2196 resulted in 75.89% nucleotide identity to IGKV3-20 germline, which could be why some of the AFS_AA/NT_ mutations (enriched based on comparison to germline) did not improve expression. In addition, AFS _AA/NT_ focuses on individual mutations and does not capture the idea of co-selected mutations in antibodies. Given that DMAbs are produced in muscle cells and not B cells, effects of tissue-specific codon usage and specific somatic mutation distributions would be valuable to further enhance expression as well as minimize variability.

Translating expression improvements into biological effect will be an important subject of future studies. This may be best demonstrated in systems with high-affinity antibodies, such as antibodies with autoimmune disease or cancer targets, whereby increased expression levels could result in a dose-sparing effect. Though we have used DNA delivery here, expression is an important parameter for all gene-encoded delivery methods. Across mRNA, DNA, and viral platforms, there is a trend for lower serum expression when scaling from small to large animals.^33^^,^^34^^,^^62^^,^^63^^,^^64^ Our methods will therefore be particularly relevant for translation to larger animal models as well as humans. As we step into a new phase of antibody delivery, the findings presented here may be critical in reducing significant barriers for antibody translation to the clinic, their broader distribution, and their concomitant clinical efficacy.

## Materials and methods

### *In vitro* protein expression and purification

All antibodies were expressed using a dual plasmid system. HC vectors contained the IgG VH leader sequence [UniProt:Q9Y298], while LC vectors contained the IGKV4-1 leader sequence [UniProt: P06312]. All vectors were codon optimized for *Homo sapiens* and/or *Mus musculus* and cloned into pVax. The relevant HC and LC pVax vectors were co-transfected into ExpiF293 cells utilizing ExpiFectamine 293 Transfection Kit (Gibco, Carlsbad, CA) as per the manufacturer’s guidelines. Supernatant was harvested 6–7 days after transfection, and antibodies were purified on a Protein A column (HiTrap MabSelect SuRe, Cytiva [Sweden]) using an AKTA Pure 25 purification system. Fractions were pooled and buffer exchanged into 1X PBS.

RBD (residues 334–527 of the SARS-CoV-2 spike glycoprotein [PDB: 6M0J]) was encoded onto pVax along with a His-tag and IgE leader sequence. Recombinant protein was purified as described previously.

### *In vivo* DMAb delivery

All animal work was performed under protocols approved by The Wistar Institute Institutional Animal Care and Use Committee (IACUC) and ACURO. Female BALB/c mice were purchased from Charles River (Malvern, PA) at 6–8 weeks old. Mice were housed in The Wistar Institute Animal Facility and studies were conducted in accordance with approved IACUC protocols. To mitigate development of mouse anti-human responses against human DMAbs,^28^^,^^65^ mice were T cell depleted with 100 μg each of recombinant anti-CD4 and anti-CD8 antibodies, dosed intraperitoneally (anti-CD4+ clone GK1.5, anti-CD8+ clone YTS 169.4, BioXCell [Lebanon, NH]) on day 0. Mice received a single injection of 50 μg of plasmid DNA (25 μg HC and 25 μg LC) co-formulated in water with hyaluronidase (12 U/injection; Sigma, St. Louis, MO) delivered to the *tibialis anterior* muscle on day 0. A 0.1-amp electric current was administered through the use of a CELLECTRA device (Inovio Pharmaceuticals, San Diego, CA) to promote plasmid uptake. Mice were bled as indicated, but generally on days 0, 7, 14, 21, 28, and 35 post DMAb injection for determination of IgG levels in sera. In some cases, a representative sample of mice was used for day 0 pre-bleeds but following DNA injection, each individual mouse was bled.

### ELISAs

ELISAs were performed using polystyrene high binding, 96-well Flat-Bottom, Half-Area Microplates (Corning, Kennebunk ME). All steps with the exception of coating were performed in 5% milk/1x PBS/0.01% Tween 20. Plates were washed with 1X PBS/0.05% Tween 20 between steps. All data were exported to Microsoft Excel and analyzed in GraphPad Prism8.

For RBD-binding ELISAs, plates were coated with 1 μg/mL anti-his antibody (Life Technologies, PA1983B, Rockford, IL) in 1X PBS for 3 h at room temperature (RT), then blocked overnight. Plates were then incubated with 10 μg/mL COV2_RBD-his for 1 h at RT. Dilutions of the relevant antibody were prepared with 8 μg/mL starting dilution, 5X dilution series, and used in duplicate, then incubated for 1 h at RT. Plates were then detected with 1:10,000 goat anti-human-Fc-HRP conjugated antibody (Bethyl A80-304P, Montgomery, TX) for 45 min, then incubated with 1-Step Ultra TMB-ELISA Substrate Solution (Thermo Scientific, Rockford, IL) for 1 min and quenched with 1 M H_2_SO_4_ sulfuric acid. Absorbance of the plates was read at 450 nM and 570 nm using a Biotek Synergy 2 plate reader. Readings were 450–570 nM normalized and blank wells were also used to subtract background absorbance.

For IgG-quantification ELISAs, plates were coated with 2.5 μg/mL anti-Fc antibody (Bethyl, A80-104A, Montgomery, TX) in 1X PBS overnight at 4°C. IgG standard (Human antibody (Whole IgG) Bethyl, P80-105 (Montgomery, TX)) was prepared (500 ng/mL starting dilution, 2X dilution series) and used in duplicate. Two wells of buffer only were also incubated as blanks. Sera was prepared with 1:50 starting dilution, 2X dilution series. Sera or IgG standard were incubated for 1 h at RT. Plates were then detected with 1:10,000 goat anti-human-Fc-HRP conjugated antibody (Bethyl, A80-304P, Montgomery, TX) for 45 min, then incubated with 1-Step Ultra TMB-ELISA Substrate Solution (Thermo Scientific, Rockford, IL) for 1 min and quenched with 1 M H_2_SO_4_. Absorbance of the plates was read at 450 nM using a Biotek Synergy 2 plate reader, and blank wells were used to subtract background absorbance. IgG standard was used to create a standard curve where IgG-quantification values could be extracted.

### Surface plasmon resonance

Kinetics of the interaction between RBD and antibody were determined using a Biacore 8k instrument (GE) and a Series S Sensor Protein A capture chip (Cytiva [Sweden]). The buffer used throughout the experiment was HBS-EP+ (3 M sodium chloride/200 mM HEPES/60 mM EDTA/1.0% Tween 20 pH = 7.6) (Teknova, Hollister, CA). Three startup cycles were used to initiate the experiment. Buffer was flowed across the chip at 10 μ L/min with 60 s contact time. Following wash, buffer was flowed across the chip at 50 μ L/min with 120s contact and 0 s disassociation time. Regeneration consisted of 10 mM glycine (pH = 1.5) flowed across the chip at 50 μ L/min, contact time 30 s. For analysis cycles, the relevant antibody was loaded to a capture level of ∼200 response units (RUs) or greater. Capture was achieved by flowing antibody across the chip at 10 μ L/min with 60 s contact time. Following wash, 4X serially diluted RBD starting at 1,000 nM was flowed across the chip at 50 μ L/min with 120 s contact and 600 s disassociation time. Regeneration was as described above. Kinetic constants were determined by 1:1 Langmuir fitting, steady state analysis, or two-state reaction fitting.

### Pseudovirus production and neutralization assay

HEK293T (ATCC) was cultured in DMEM supplemented with 10% fetal bovine serum (FBS) and 1% penicillin-streptomycin (P/S) antibiotic at 37°C/5% CO2 (complete DMEM). HEK293T cells were transfected with a 1:1 ratio of pNL4-3.luc.R-E− backbone (Aldevron, Parkway, ND) and a plasmid encoding the spike protein of SARS-CoV-2 (USA- WA1/2020) (Genscript, Piscataway, NJ) using GeneJammer (Agilent, Chicago, IL). Transfection supernatants were collected after 48 h, filtered and stored at −80°C.

For pseudovirus neutralization assays, HuCHOAce2 cells (Creative Biolabs, Catalog No. VCeL-Wyb019, Shirley, NY) were plated in 96-well plates at 10,000 cells/well and incubated for 24 h at 37°C/5% CO2. In-house produced recombinant antibody for all 2196 antibodies of interest were serially diluted (starting dilution 1:50, 5-fold dilution series) in complete DMEM and incubated with pseudovirus for 90 min in duplicate, then were transferred to the plated HuCHOAce2 cells. Following 72 h of incubation at 37°C/5% CO2, plates were developed using BriteLite plus luminescence reporter system (PerkinElmer, Waltham, MA) and luminescence was read using a Biotek Synergy plate reader. Neutralization titers (median infective dose [ID_50_]) were calculated as the reciprocal dilution that yielded a 50% reduction in luminescence compared with virus-only control wells. Background subtraction of luminescence from cells-only wells was also applied. ID_50s_, in combination with antibody concentration (μg/mL), were additionally used to calculate inhibitory concentrations (IC_50s_ = antibody concentration/ID_50_). These show the potency of a given antibody while also providing control for variable starting concentrations.

### Structural prediction and analysis

The structure of 2196 was extracted from the PDB (PDB: 7L7E)^29^ and visualized in Pymol 2.0.

The structures of 2196 HC paired with alphamod1 LC, 2072 LC, or rationally designed variant LCs were predicted using ColabFold, an accessible version of AlphaFold2.^39^^,^^40^ Predictions did not include templates. Five structures were predicted and the top-ranked structure was loaded into Pymol 2.0 for visualization.

### OAS searching and antibody frequency scores

Unpaired LC sequences from the OAS^47^^,^^48^ were downloaded from https://opig.stats.ox.ac.uk/webapps/oas/oas. To avoid bias in antibody germlines that might be caused by exposure to antigen or natural biases from autoimmune or other types of conditions, sequences were filtered by disease state: none. Duplicates were excluded from the search. This resulted in a searchable database of approximately 291 million unpaired LC sequences obtained from healthy donors. Searches for a particular antibody germline were specified by gene locus, gene segment, and gene family, but not by allele.

When incorporating particular sequence motifs into a search, regular expressions were employed. Full-length antibody searches could be specified by V and J gene and a regular expression was used to search for relevant sequences and to restrict sequences to a specific length.

For CDRL1, 2, and 3 length distribution, IGKV3-20 was specified as the germline gene. Anchoring positions for each regular expression search were defined as outlined in North et al.^66^ In particular, CDRL1 sequences were searched using [G][A-Z][5][S,T,N][C] …. [W][Y,F,L,V][Q,L] as the anchoring motifs. For CDRL2, [L,W,V][I,V,M] … [G][V,I][P,S] was used, and for CDRL3 [Y][A-Z][1][C] … [F][G][A-Z][1][G][T] served as the anchoring motif. These slightly more restrictive definitions for CDRs were employed to avoid mis-identification of CDRL1 and CDRL3s as some of the anchoring residues for these two CDRs are very similar. For CDRL1, 2, and 3 length distribution, IGKV3-20 was specified as the germline gene.

For development of AFS_AA_/AFS_NT_, regular expressions were again employed, using the same anchoring positions around the CDRs. Hits from each search were then collated into a single FASTA file, which was then processed in Python to calculate the frequency of every amino acid at every single position in the search. For example, for a given position in a searched sequence, the relative frequency that A, C, D, E, F, G, H, I, K, L, M, N, P, Q, R, S, T, V, W, or Y occurred across the collated hits was calculated.

Frequency data were then exported to a CSV file for further processing. Excel macros were used to sort each position in order of relative frequency and to compare frequencies of each amino acid to the germline antibody sequency. Germline % was calculated as the 100∗(frequency of the germline amino acid at a given position). The non-germline % score was calculated as 100∗(frequency of non-germline amino acid)/(1-frequency of germline amino acid). The non-germline percent score of each amino acid was compared. A delta frequency score was used to determine enrichment of a particular non-germline amino acid by comparing relative preference for each amino acid at a given position. Specifically, the delta score was tabulated as (highest % non-germline amino acid – second highest % non-germline amino acid). No score was calculated if germline was not the most prevalent amino acid.

Frequency score could be skewed by the likelihood of a particular codon change from the germline codon. For example, enrichment of a particular amino acid could simply be due to several 1-nucleotide changes from the germline sequence all encoding for that same amino acid, indicating enrichment due to random chance. To normalize for this possibility, the frequency of each amino acid was divided by the number of 1-nucleotide changes from the germline sequence that could encode for that amino acid. Applying this to every amino acid yielded AFS_AA_.

The development of a codon-based version of AFS (AFS_NT_) followed a similar workflow, but instead of considering amino acid frequencies, the frequency of each specific codon was calculated across the entire V-gene. This was converted into a percent non-germline score, and AFS_NT_ was tabulated as (highest % non-germline codon – second highest % non-germline codon). If the most frequently observed non-germline codon also coded for germline amino acid, no AFSNT score was calculated since this would suggest that germline was the preferred amino acid at that position.

### OAS V-gene family distribution

In order to treat all sequences the same way, the total number of identifiable LC sequences was determined by performing a search on the downloaded LC repertoire and specifying the V-gene family. Each hit (which corresponds to a single sequence in the repertoire) was then collated into a single FASTA file and the number of total sequences per V-gene family was determined from this. Counts are expressed in millions of sequences.

### OAS donor count

To understand the contribution to AFS scores on a per donor basis, donor counts were obtained. Following a given search on the OAS, data were collated into a single file to extract total number of sequence counts, and subset sequences by donor name. Counts by donor were extracted, then graphed using Prism 8.

## Data and code availability

Data containing the frequencies of amino acids and nucleotides per position that made up the basis of AFS_AA_ and AFST_NT_ are available upon request for all LC germlines referenced. All other data generated/analyzed are contained within this article and supplemental information.

## Acknowledgments

We thank the Animal Facility staff at the Wistar Institute for providing animal care. The graphical abstract and some portions of figures were created with BioRender (BioRender.com). This research was, in part, funded by the US government, 10.13039/100000185Defense Advanced Research Projects Agency DARPA awards HR0011-21-9-0001 and N660012014049. The views and conclusions contained in this document are those of the authors and should not be interpreted as representing the official policies, either expressed or implied, of the US government. Approved for Public Release, Distribution Unlimited. Research reported in this publication was supported by the National Center for Advancing Translational Sciences of the 10.13039/100000002National Institutes of Health under award number TL1TR001880. The content is solely the responsibility of the authors and does not necessarily represent the official views of the National Institutes of Health.

## Author contributions

M.H., D.W.K., and A.P. conceived ideas, designed research, and wrote and edited the paper. M.H., J.C., K.F., A.R.T., and A.P. performed research. A.H., A.K., and N.S. produced and purified proteins. C.E.H. and E.N.G. completed pseudoneutralization assays. S.S. performed OAS donor analysis. E.M.P. and D.B.W. contributed new reagents/analytic tools.

## Declaration of interests

The authors have multiple pending patents in the area of DMAb technology. D.B.W. has received grant funding, participates in industry collaborations, has received speaking honoraria, and has received fees for consulting, including serving on scientific review committees and board series. Remuneration received by D.B.W. includes direct payments and stock or stock options. D.B.W. also discloses the following paid associations with commercial partners: GeneOne (consultant), Geneos (advisory board), AstraZeneca (advisory board, speaker), Inovio (BOD, SRA, Stock), Sanofi (advisory board), and BBI (advisory board).

## Supplemental information

Document S1. Figures S1–S19

Document S2. Article plus supplemental information
