# Supplementary material for: Structure and sequence engineering approaches to improve in vivo expression of nucleic acid-delivered antibodies
Source: Mol Ther. 2024 Nov 19;33(1):152–67. doi: 10.1016/j.ymthe.2024.11.030 (PMC11764276; doi:10.1016/j.ymthe.2024.11.030)
Supplement: Document S1. Figures S1–S19 [file mmc1.pdf]

YMTHE, Volume *33*

Supplemental Information

Structure and sequence engineering approaches to improve *in vivo* expression of nucleic acid-delivered antibodies

Michaela Helble, Jacqueline Chu, Kaitlyn Flowers, Abigail R. Trachtman, Alana Huynh, Amber Kim, Nicholas Shupin, Casey E. Hojecki, Ebony N. Gary, Shahlo Solieva, Elizabeth M. Parzych, David B. Weiner, Daniel W. Kulp, and Ami Patel

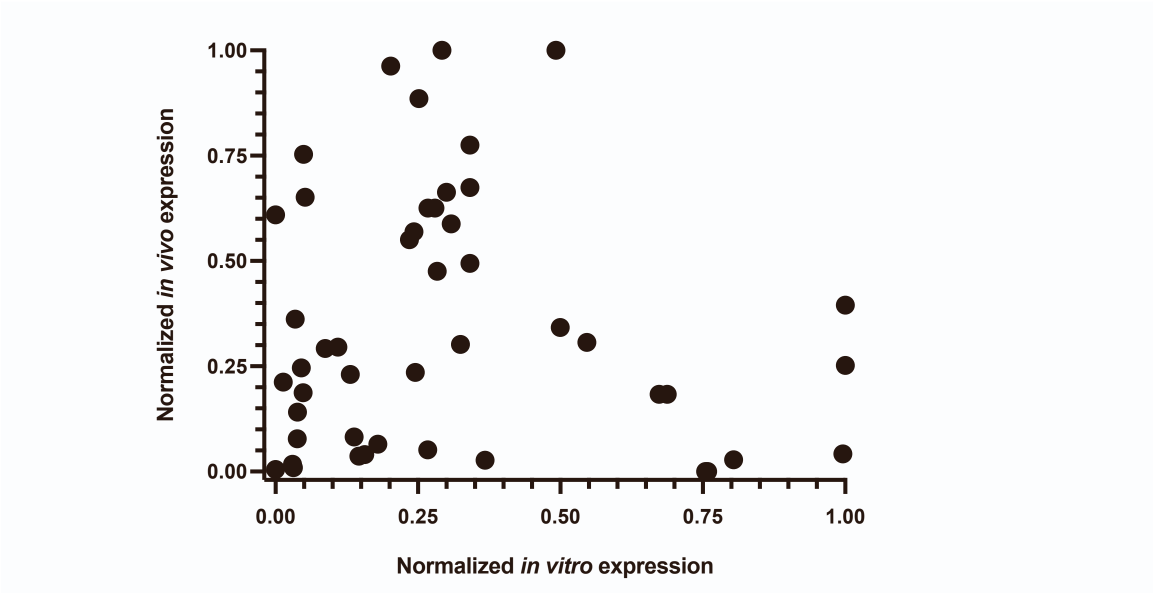

Figure S1. *In vivo* vs *in vitro* expression of assorted DMAbs. Forty-seven DMAbs, comprised of different VH/VL genes and different target antigens, were expressed *in vitro* in ExpiF293 cells and purified on a protein A column. The same DMAbs were expressed in female BALB/c mice and serum concentration was determined through quantification ELISA. Normalized *in vitro* and *in vivo* expression was calculated for the set of 47 DMAbs.

A

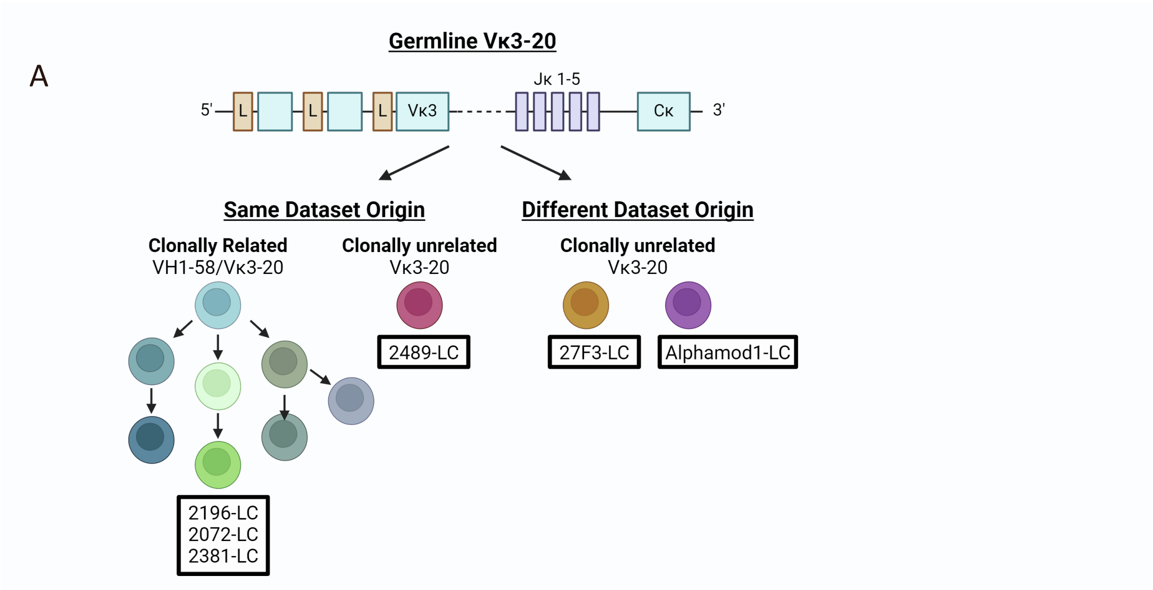

B

```
                                    10         20         30         40         50         60
Germline_LC (VK3-20)  EIVLTQSPGT LSLSPGERAT LSCRASQSVS SSYLAWYQQK PGQAPRLLIY GASSRATGIP
2196_LC               EIVLTQSPGT LSLSPGERAT LSCRASQSVS SSYLAWYQQK PGQAPRLLIY GASSRATGIP
2072_LC               EIVLTQSPGT LSLSPGERAT LSCRASQSVS SSYLGWYQQK PGQAPRLLIY GASSRATGIP
2381_LC               EVVLTQSPGT LSLSPGERAT LSCRASQSVS SSYLAWYQQK PGQAPRLLIY GASSRATGIP
27F3_LC               EIVLTQSPGT LSLSPGERAT LSCRASQSVS SSYLAWYQQK PGQAPRLLIY GASSRATGIP
2489_LC               EIVLTQSPGT LSLSPGERAS LSCRASQSVS SSYLAWYQQK PGQAPRLLIY GASSRATGIP
Alphamod1_LC          EIVLTQSPGT LSLSPGERAT LSCRASQTIS NNFVAWYQQK PGQAPRLLIY GASTRATGIP

                                    70         80         90        100
Germline_LC (VK3-20)  DRFSGSGSGT DFTLTISRLE PEDFAVYYCQ QYGSSP-WTF GQGTKVEIK
2196_LC               DRFSGSGSGT DFTLTISRLE PEDFAVYYCQ HYGSSRGWTF GQGTKVEIK
2072_LC               DRFSGSGSGT DFTLTISRLE PEDFAVYYCQ QYGSSP-WTF GQGTKVEIK
2381_LC               DRFSGSGSGT DFTLTISRLE PEDFAVYHCQ HFGSSSQWTF GQGTKVEIK
27F3_LC               DRFSGSGSGT DFTLTITRLE PEDFAVYYCQ QYVSTP-LTF GGGTKVEIK
2489_LC               DRFSGSGSGT DFTLTISRLE PEDFAVYYCQ QYGSSP-FTF GGGTKVEIK
Alphamod1_LC          DRFSGSGSGT DFTLTISRLE PEDFAVYYCQ QYGSSP-YTF GQGTKLEIK
```

Figure S2. Chain-swapped IGKV3-20 light chains. A) Overview of sequences selected for chain swapping experiments. Light chains originating from the same dataset all target SARS-COV-2, though only some are derived from the same clone (clonally related). Clonally unrelated clones from different datasets target alternative antigens B) Sequences of chain-swapped LCs. All IGKV3-20 germline LCs used in the chain-swap experiments, both clonally related and unrelated, were aligned using IGKV3-20 germline as reference. Mutations from germline are highlighted.

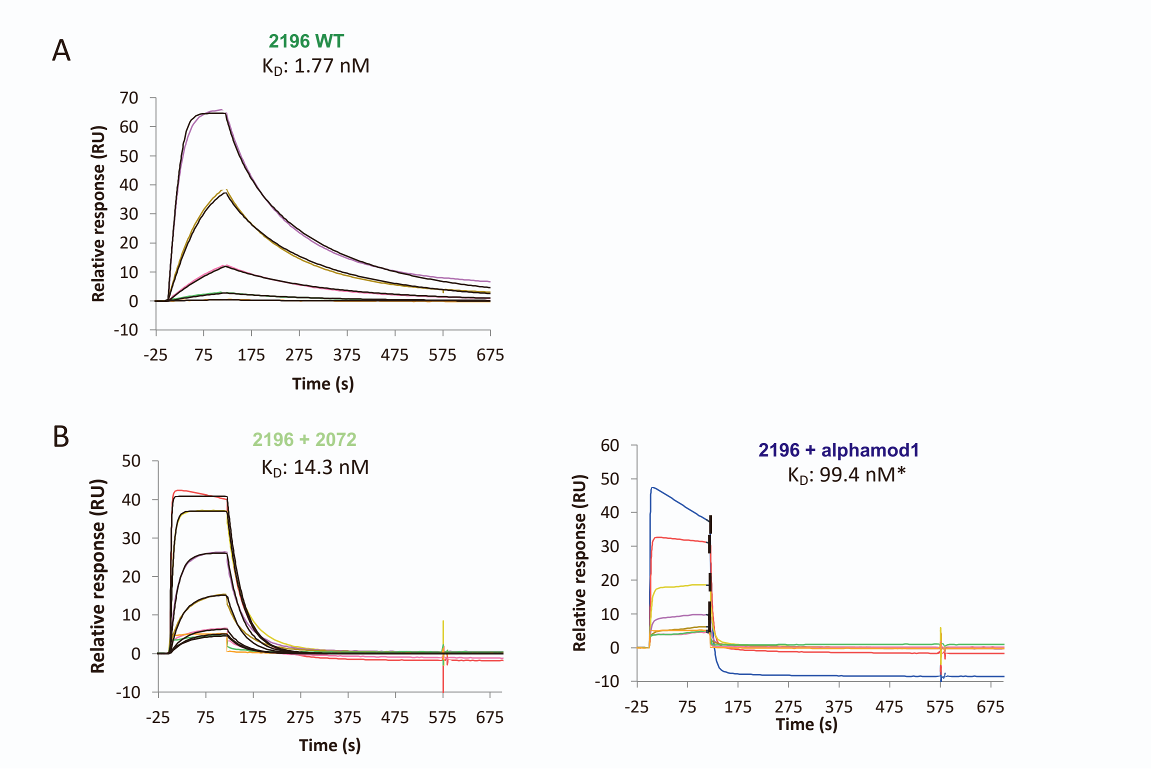

Figure S3. SPR binding kinetics of top-expressing chain swap variants to WT RBD. **A)** 2196 WT or **B)** Top-expressing chain-swapped antibodies were produced in ExpiF293 cells and purified on a protein A column. Binding to RBD was determined through either 1:1 Langmuir fitting, or *steady state affinity. Thick black tick marks indicate that point of the curve that was used for steady state affinity calculations.

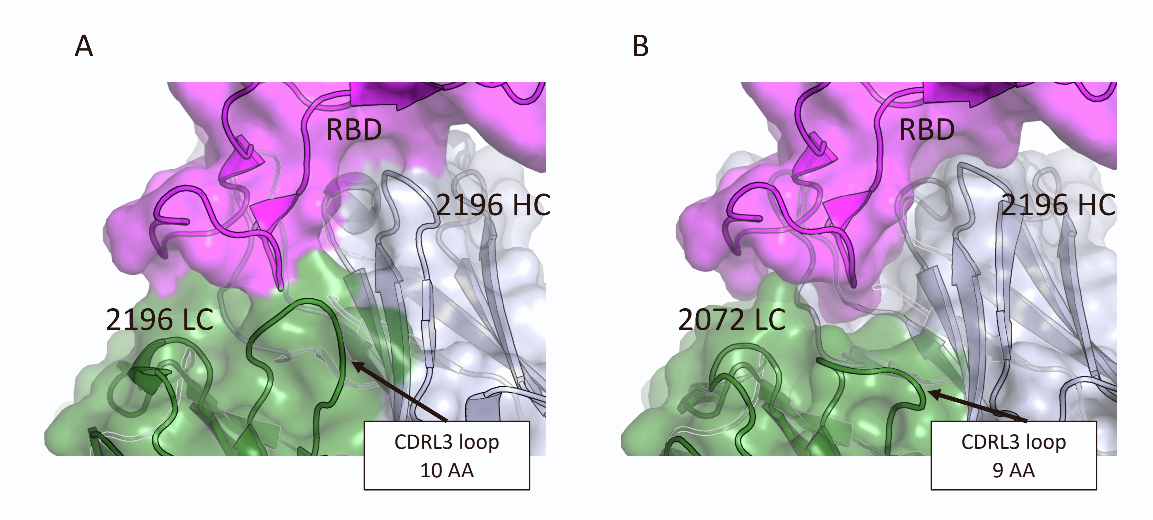

Figure S4. CDRL3 loop length comparison. Surface models of **A)** 2196 HC + 2196 LC (PDB: 7L7E) or **B)** 2196 HC + 2072 LC (Alphafold2 model). The CDRL3 loop of 2072 and alphamod1 are identical, and one amino acid shorter than the CDRL3 loop of WT 2196, removing a 'hugging' effect around the RBD that might impact binding affinity.

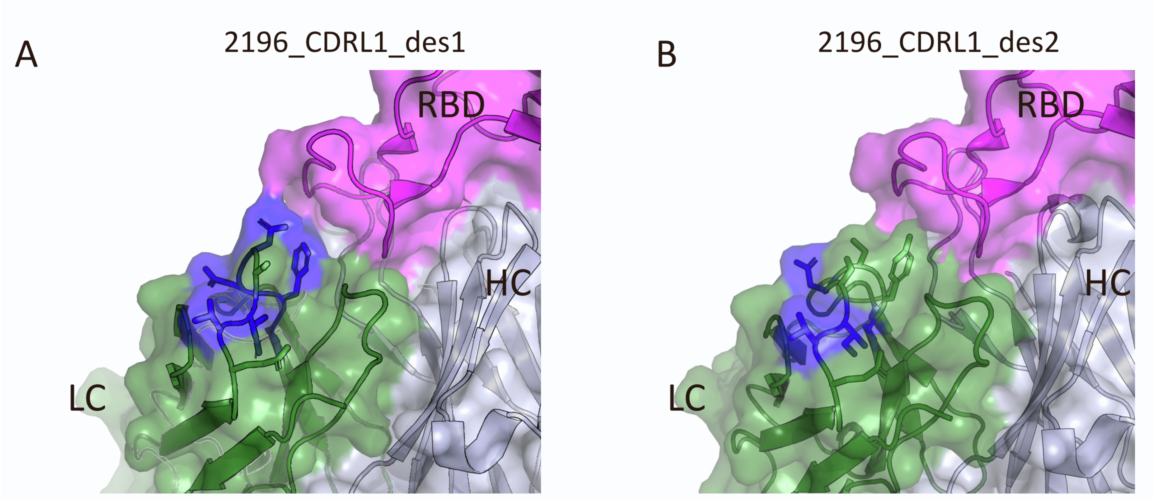

Figure S5. CDRL1-focused rational design models. AlphaFold2 surface models of **A)** 2196_CDRL1_des1 and **B)** 2196_CDRL1_des2. Amino acids highlighted in blue are mutated from 2196 WT. Side chains are shown as sticks for CDRL1 residues.

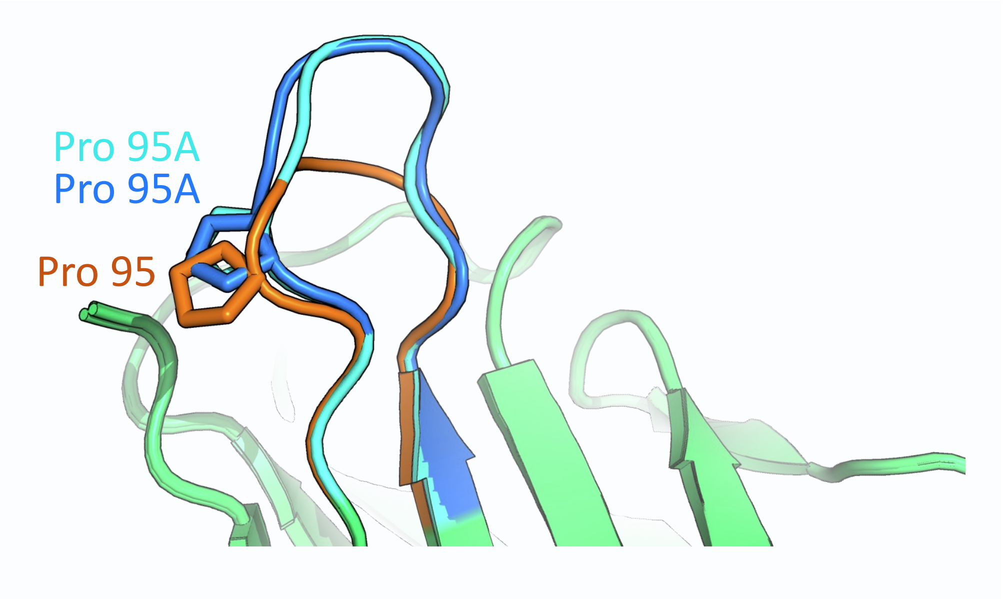

Figure S6. Proline alignment of CDRL3 rational design variants. Alphafold2 models of CDRL3 variants. Light chain is shown in green, heavy chain is omitted for viewing clarity. CDRL3 loops are colored by variant: 2196_CDRL3_P (orange), 2196_CDRL3_RP (blue) and 2196_CDRL3_GP (cyan). The proline side chains are shown, and align spatially, despite being at sequentially different locations in the LC. The proline is at position 95 in 2196_CDRL3_P, and at position 95A for 2196_CDRL3_RP and 2196_CDRL3_GP.

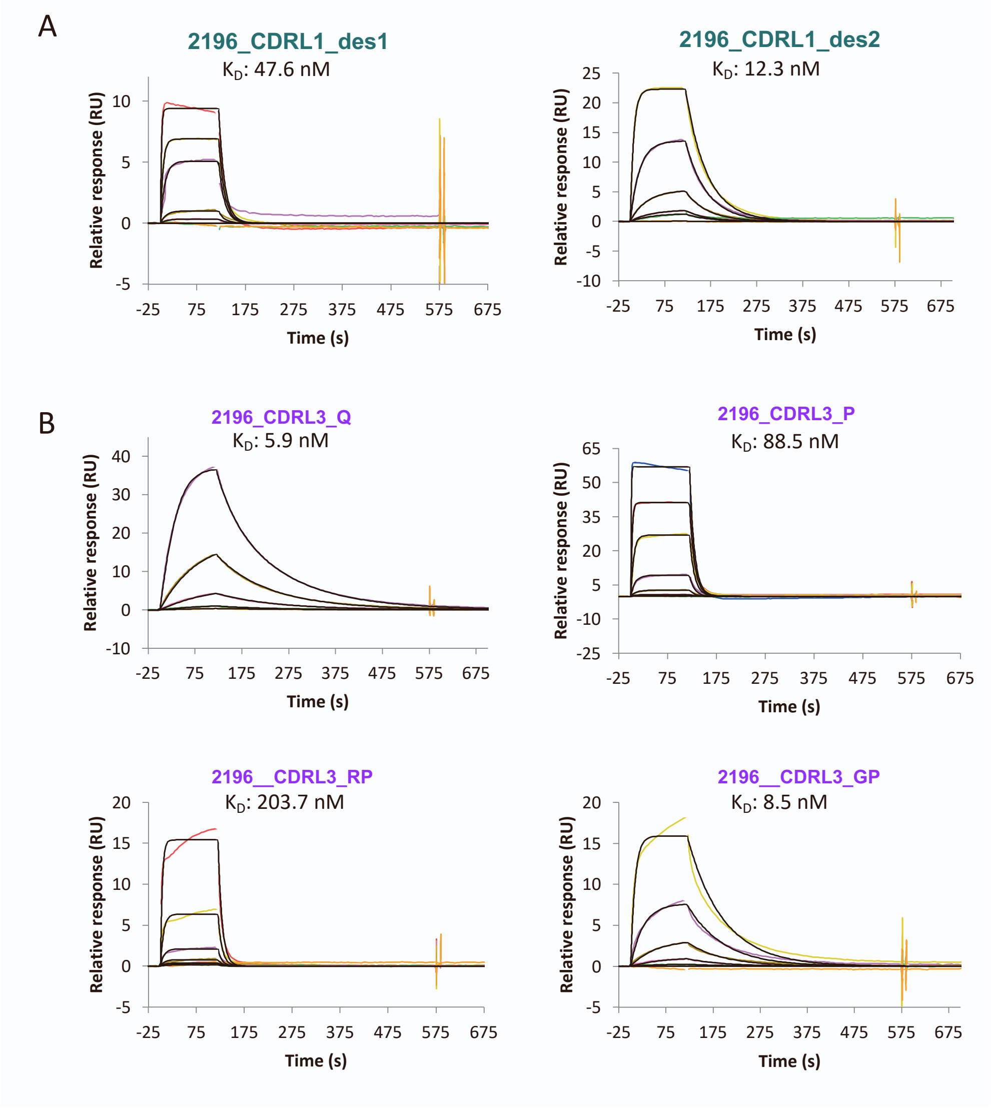

Figure S7. SPR binding kinetics of rational design variants to WT RBD. All indicated antibodies were produced in ExpiF293 cells and purified on a protein A column, and binding to WT RBD was then determined by SPR. 1:1 Langmuir fitting was used. **A)** CDRL1-based designs **B)** CDRL3-based designs.

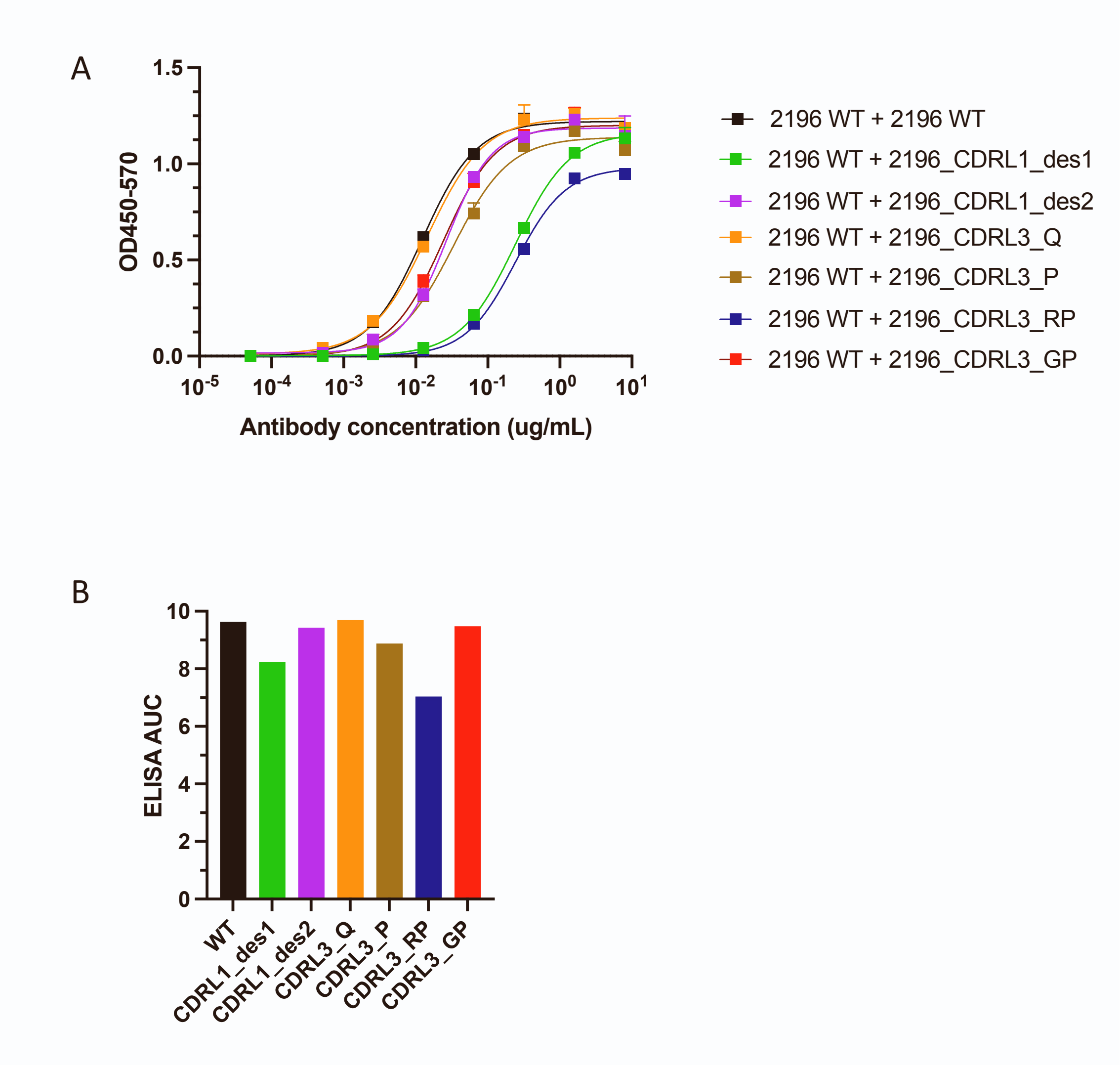

Figure S8. Binding of rationally designed variants to WT RBD. A) Binding of CDRL1 and CDRL3 rational design antibody variants to WT RBD as determined by ELISA. Plates were coated with 1ug/mL anti-his followed by incubation with 10ug/mL WT RBD. The relevant antibody was then serially diluted and incubated at the concentrations indicated, in duplicate. Mean and error shown **B)** AUC. AUC values were calculated from the ELISA in A) using Prism 8.

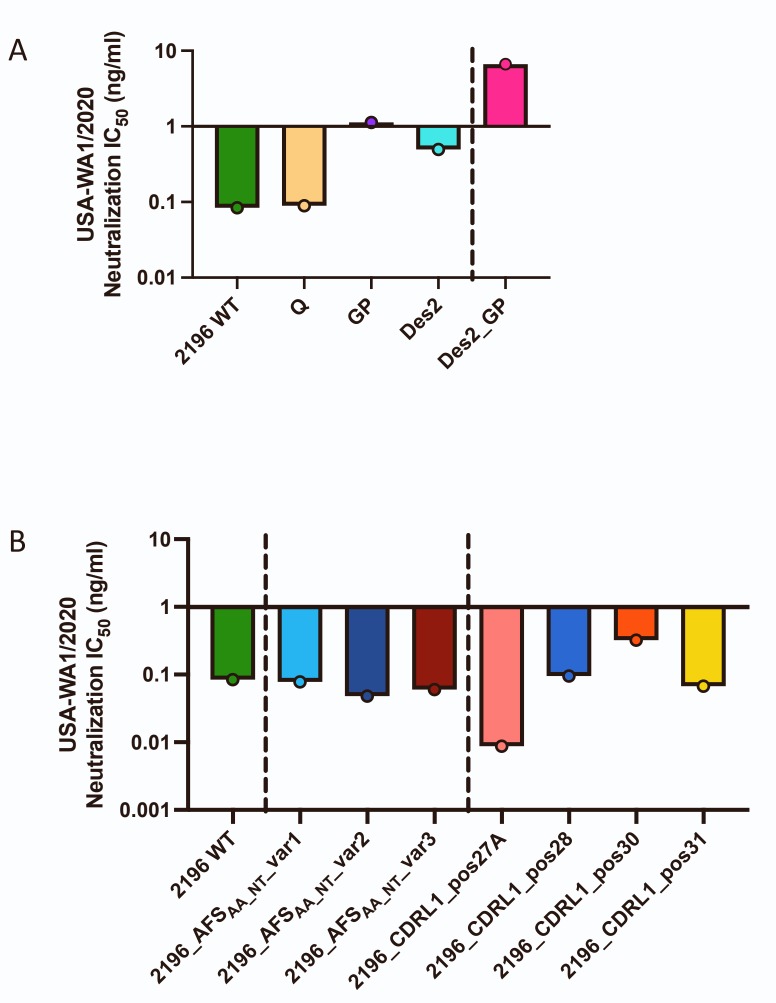

Figure S9. Pseudotyped SARS-CoV-2 Neutralization. IC_{50} against pseudotyped USA-WA1/2020 was determined for recombinant antibodies designed from **A)** CDRL1- and CDRL3- focused rational design efforts and **B)** AFS_{AA} and AFS_{NT} scores, including single mutant CDRL1 scanning variants.

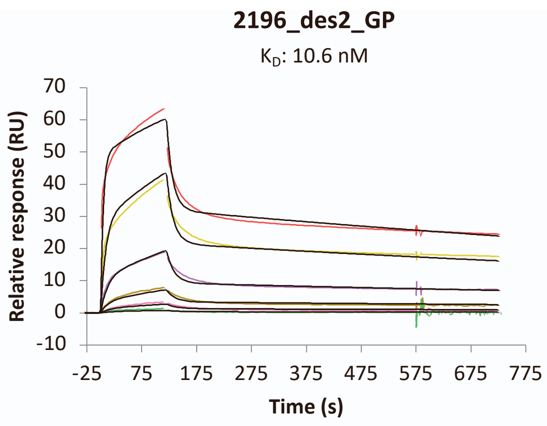

Figure S10. SPR binding kinetics of combined variant 2196_des2_GP to WT RBD. The antibody was produced in ExpiF293 cells and purified on a protein A column. Binding to WT RBD was then determined by SPR. Two state reaction fitting was used.

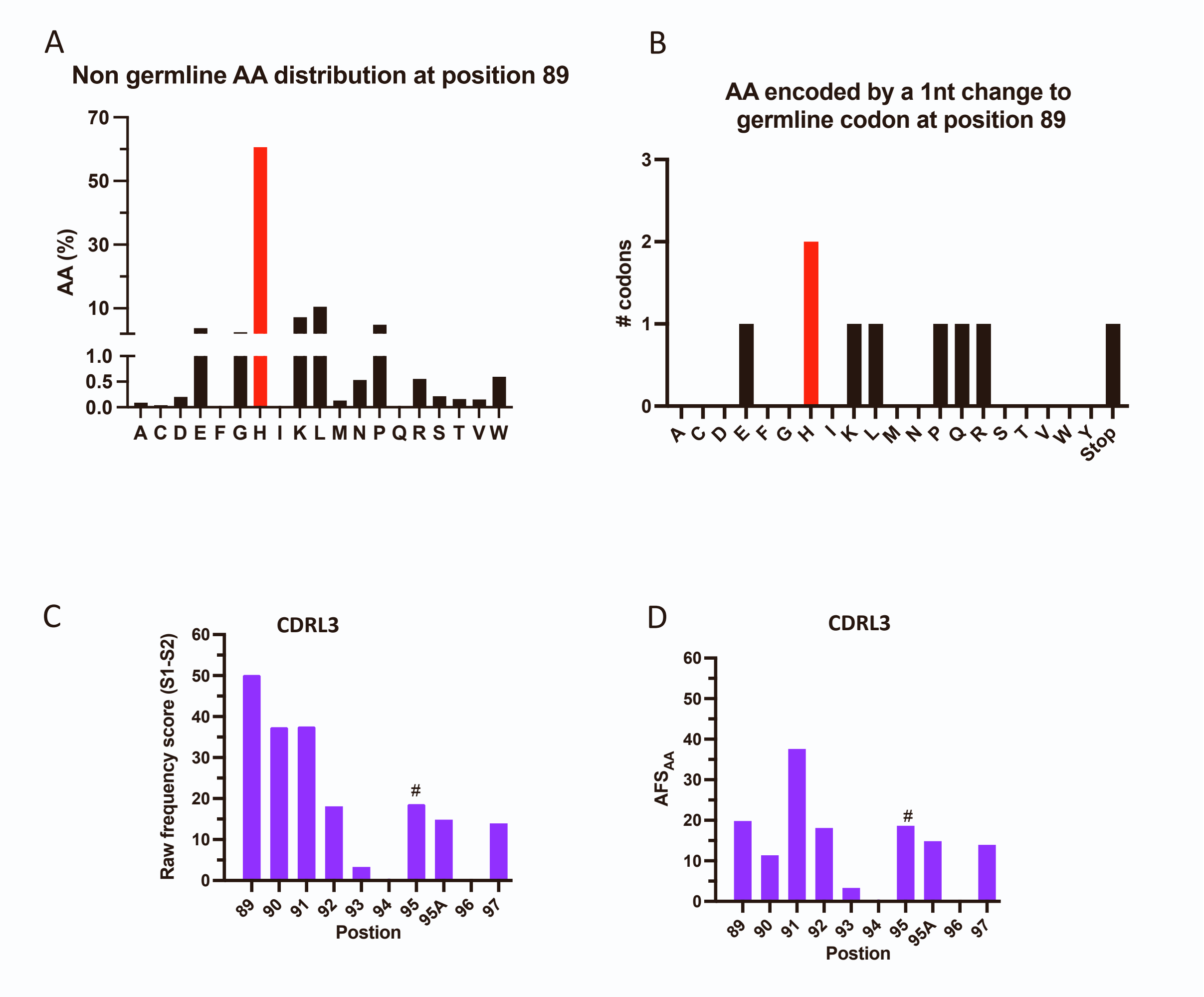

Figure S11. Antibody frequency score refinement. **A)** Non-germline amino acid frequency at position 89 in the CDRL3. The most frequent non-germline amino acid (H) is colored red. **B)** Amino acids encoded by a single nucleotide change to the germline codon. Germline codon is CAG, and there are 9 possible single nucleotide changes. Two of these nine encode for a histidine, which is also the most frequent non-germline amino acid at position 89. **C)** Delta frequency score of CDRL3. The non-adjusted frequency score is S1-S2 at every position, with no codon bias taken into account. #VJ junction. **D)** Scaled frequency score of CDRL3 (AFS_{AA}). Scores are adjusted by the number of 1-nt change codons encoding for the most prevalent amino acids, to account for the possibility that an observed enrichment is due to random chance rather than selection. #VJ junction.

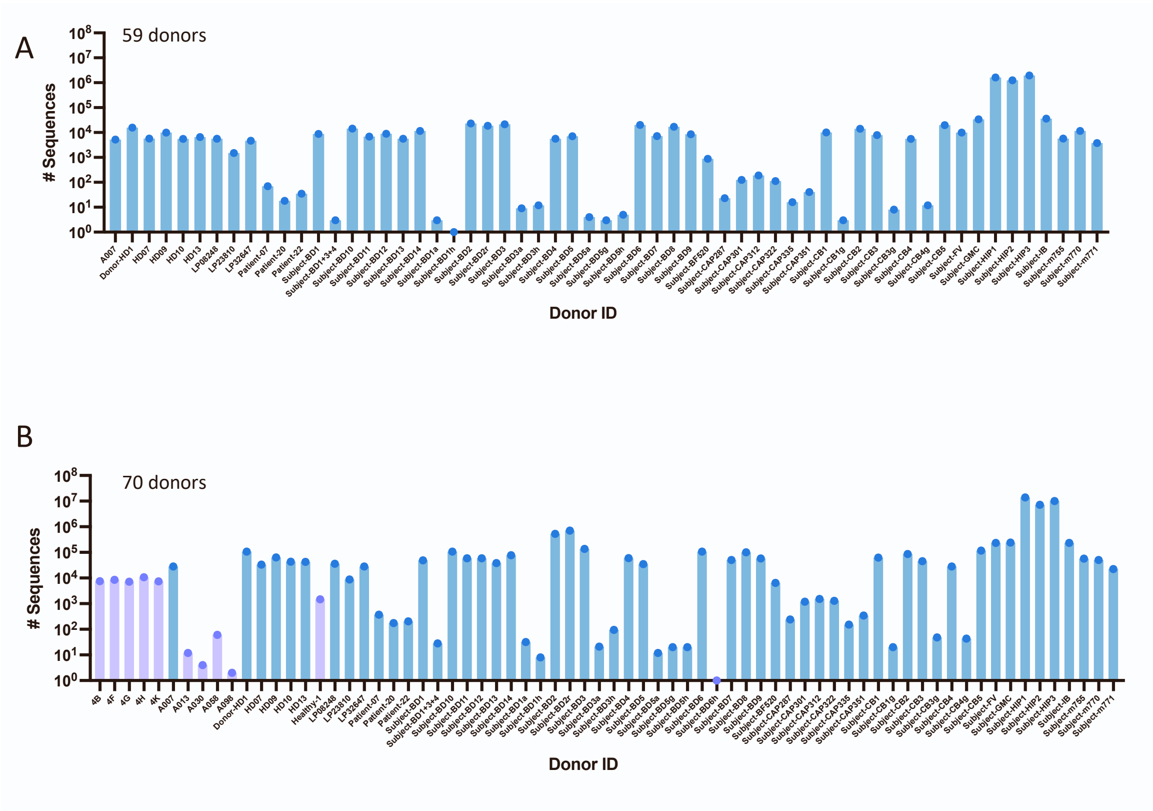

Figure S12. **IGKV3-20 donor sequences**. **A)** Contributing donors and sequences for 2196-like IGKV3-20 antibodies. Full length 2196-matched (identical CDR length) IGKV3-20 antibodies were sorted by donor and the total number of sequences contributed by each individual donor was summed. **B)** Contributing donors and sequences for any IGKV3-20 antibodies. No CDR length restrictions were imposed. Antibodies were sorted by donor and the total number of sequences contributed by each individual donor was summed. Purple bars indicate donors absent from A) that do not have any 2196-like antibody sequences in their repertoires.

```
                        10         20         30         40         50         60
IGKV320*01    EIVLTQSPGT LSLSPGERAT LSCRASQSVS SSYLAWYQQK PGQAPRLLIY GASSRATGIP
IGKV320*02    EIVLTQSPAT LSLSPGERAT LSCRASQSVS SSYLAWYQQK PGQAPRLLIY GASSRATGIP
IGKV3D20*01   EIVLTQSPAT LSLSPGERAT LSCGASQSVS SSYLAWYQQK PGLAPRLLIY DASSRATGIP

                        70         80         90
IGKV320*01    DRFSGSGSGT DFTLTISRLE PEDFAVYYCQ QYGSSP
IGKV320*02    ARFSGSGSGT DFTLTISRLE PEDFAVYYCQ QYGSSP
IGKV3D20*01   DRFSGSGSGT DFTLTISRLE PEDFAVYYCQ QYGSSP
```

Figure S13. IGKV3-20 germline alleles. IGKV3-20 germline sequences were aligned using IGKV3-20*01 as the reference sequence. Allelic differences are highlighted. These positions and amino acids do not correspond to positions and amino acids selected for in AFS. Positions are numbered sequentially rather than by Kabat.

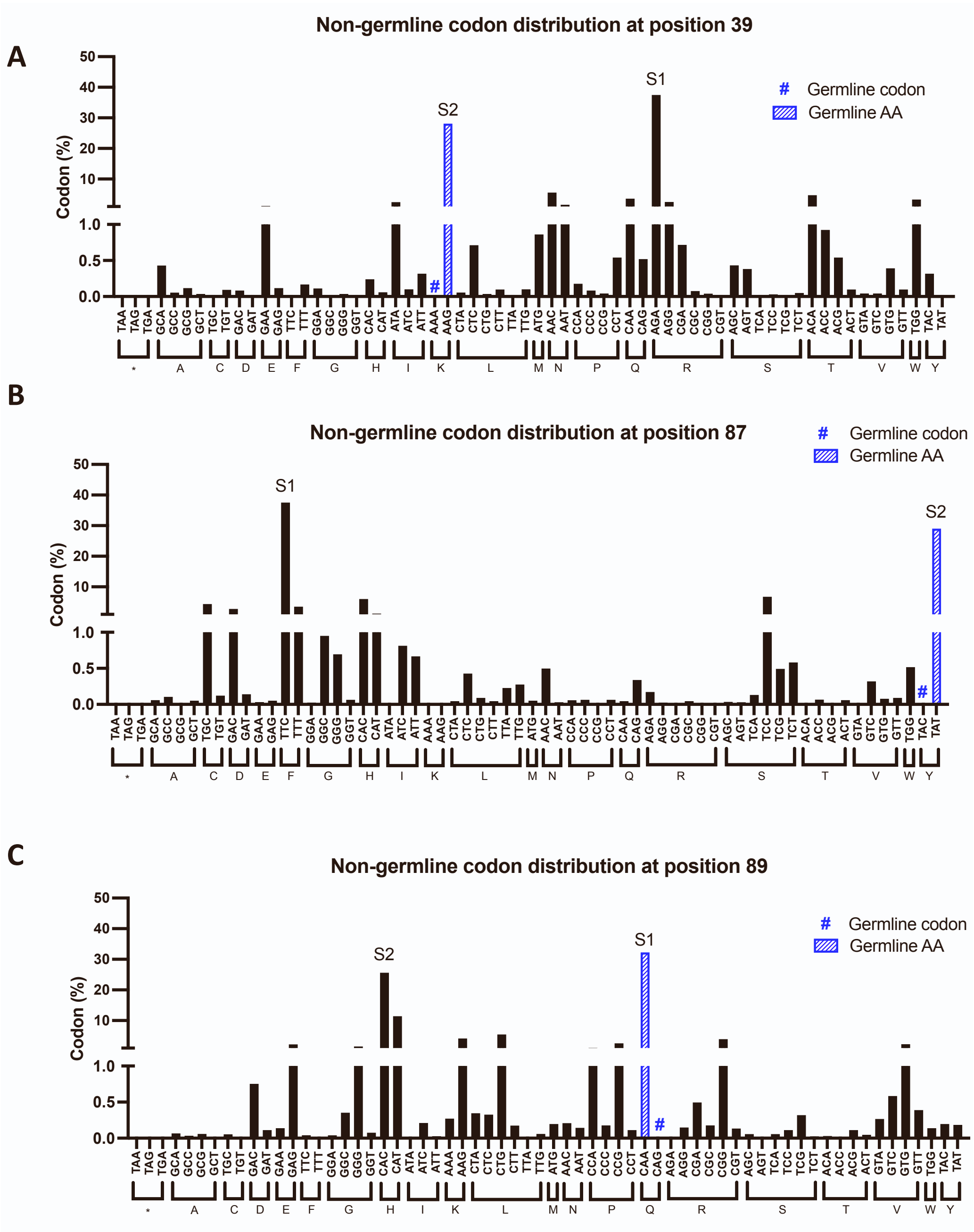

Figure S14. Preference for germline amino acid. The non-germline codon distribution at positions A) 39, B) 87 and C) 89. **A)** At position 39, the germline amino acid is K (AAA). S2 is a silent mutation in which an alternative germline codon (AAG) has high preference. **B)** At position 87, the germline amino acid is Y (TAC). S2 is a silent mutation in which an alternative germline codon (TAC) has high preference **C)** At position 89, the germline amino acid is Q (CAG). Here, S1 is a silent mutation in which an alternative germline codon (CAA) is the highest frequency, suggesting that the germline amino acid is preferred. Positions like this are excluded from overall AFS_{NT} scoring due to this germline preference.

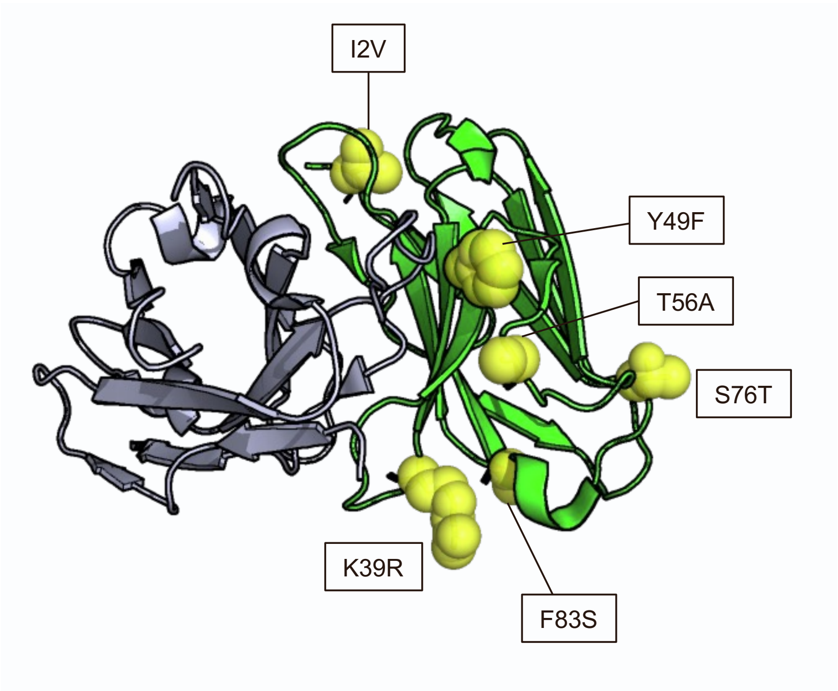

Figure S15. Location of new AFS_{AA} and AFS_{NT} derived mutations. New mutations from AFS_{AA}_NT_var1-3 were mapped onto the structure of 2196 (PDB:7L7E) to determine if the new amino acids suggested by AFS scores might cause HC:LC pairing clashes. Pymol's mutagenesis wizard was used to introduce the AFS-suggested mutations, and side chain atoms for each new mutant are shown in yellow spheres.

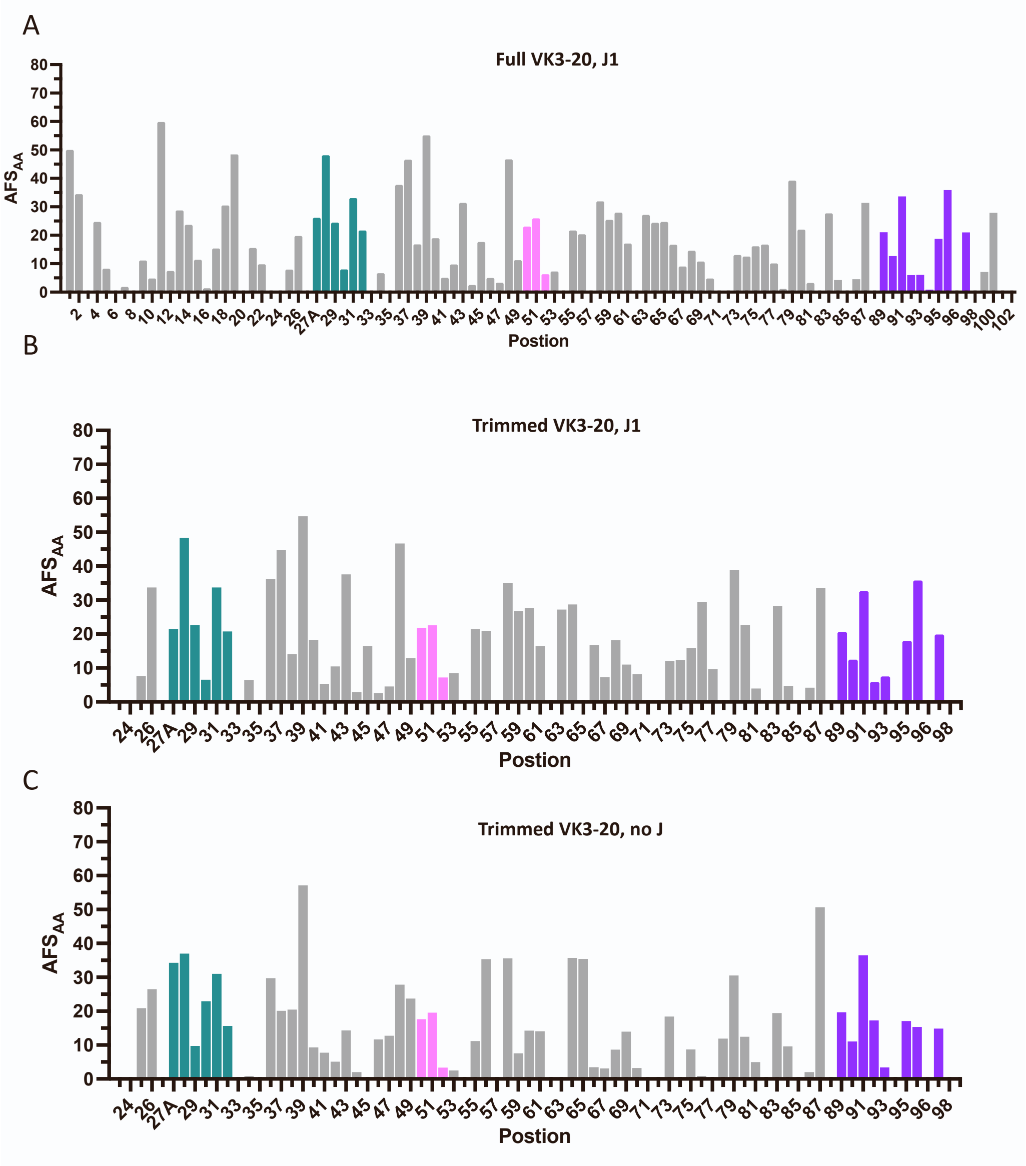

Figure S16. Alternative OAS search terms and corresponding AFS$_{AA}$. All AFS scores were developed as described in Figure 4. They are S1-S2 per position, and all include codon adjustment to the delta frequency score. These AFS scores only differ in the search terms defined in the OAS to extract amino acid frequencies per position. **A)** The full V gene was searched, with additional criteria that the J gene be matched to 2196's J gene (J1). All antibodies represented here are both IGKV3-20 and J1 **B)** The search motif was trimmed to search from anchoring positions at the CDRL1 to anchoring positions at the CDRL3. This was done to ensure that antibodies missing their first few amino acids due to less robust sequencing were captured in the search. The J gene was specified as additional criteria. All antibodies represented here are both IGKV3-20 and J1. **C)** The search motif was trimmed as in B, but here, the J gene was excluded to not restrict antibodies by two gene usage criteria.

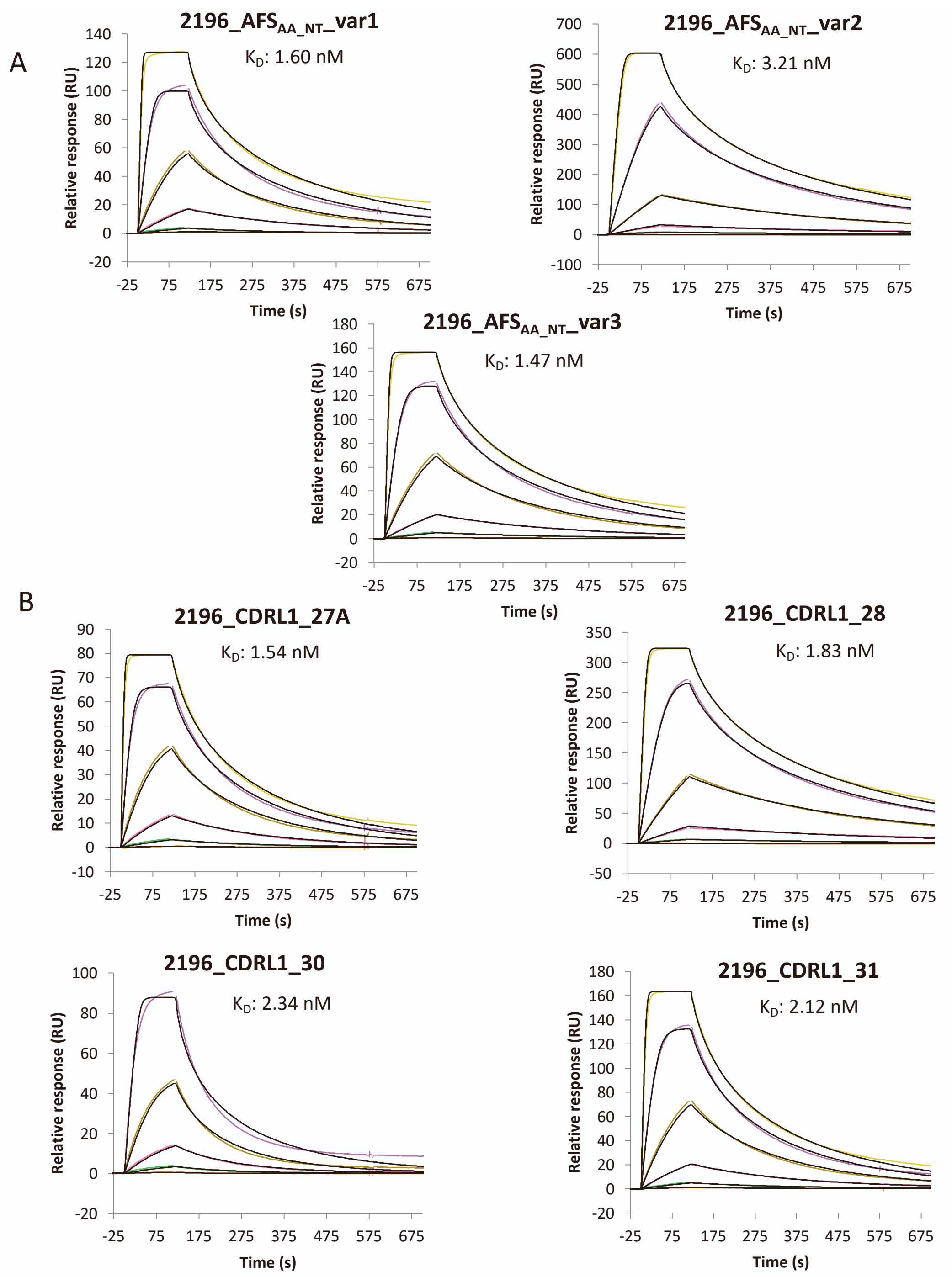

Figure S17. SPR binding kinetics of AFS_{AA} and AFS_{NT} variants to WT RBD. All indicated antibodies were produced in ExpiF293 cells and purified on a protein A column. SPR was used to determine binding to RBD. K_Ds were determined by 1:1 Langmuir fitting. SPR curves and K_Ds for **A)** variants determined by AFS_{AA} and AFS_{NT} scores or **B)** single mutation CDRL1 variants.

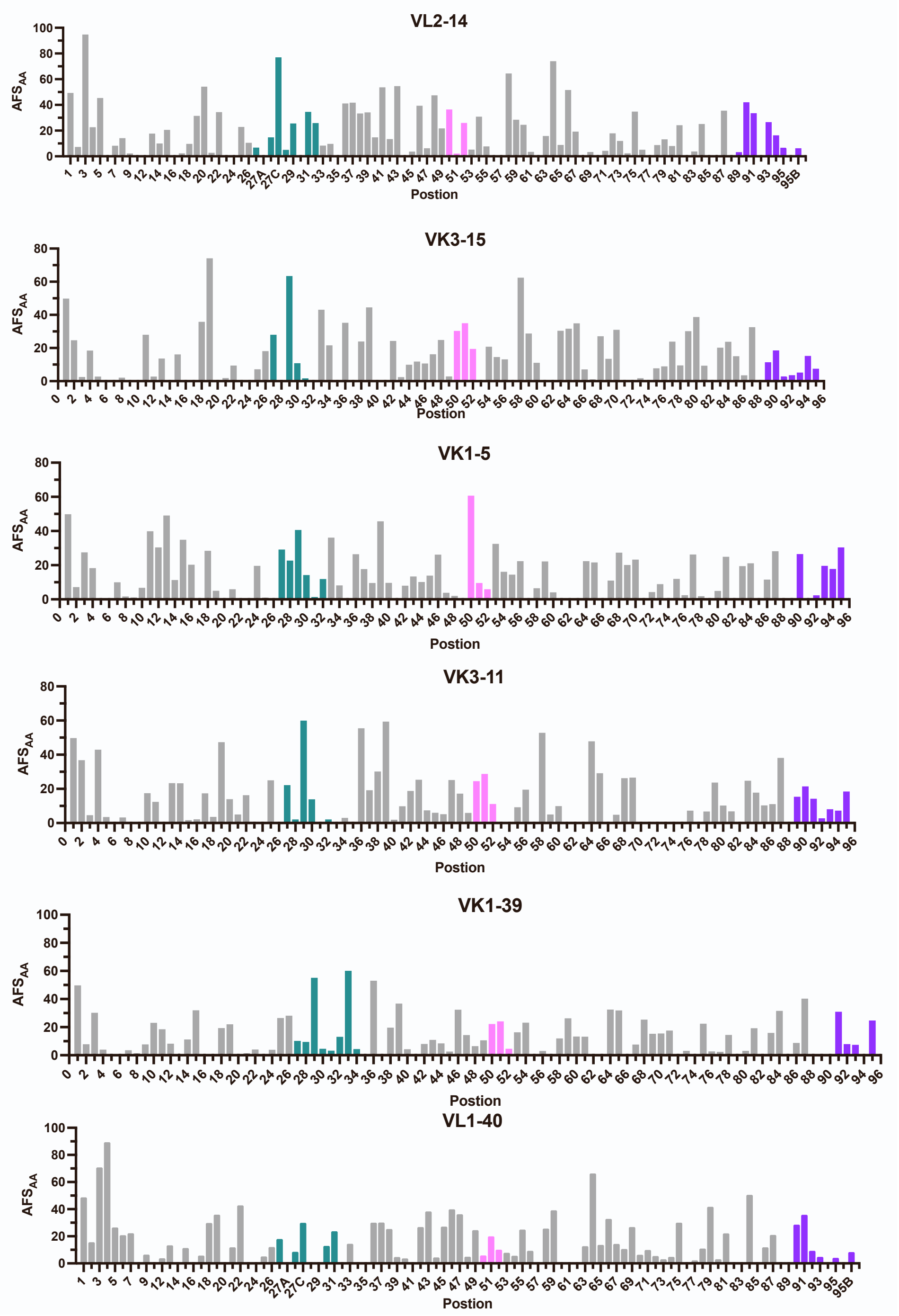

Figure S18. AFS_{AA} for most common light chain germlines. AFS_{AA} scores for the most common V-gene germlines in the OAS (IGLV2-14, IGKV3-15, IGKV1-5, IGKV3-11, IGKV1-39, IGLV1-40). All AFS_{AA} scores were developed as described in Figure 4. They are S1-S2 for amino acids per position, and all include codon adjustment to the delta frequency score. The full V gene was searched with germline encoded CDRL1 and CDRL2 lengths, but length was allowed to vary for CDRL3 to capture more antibody sequences of interest.

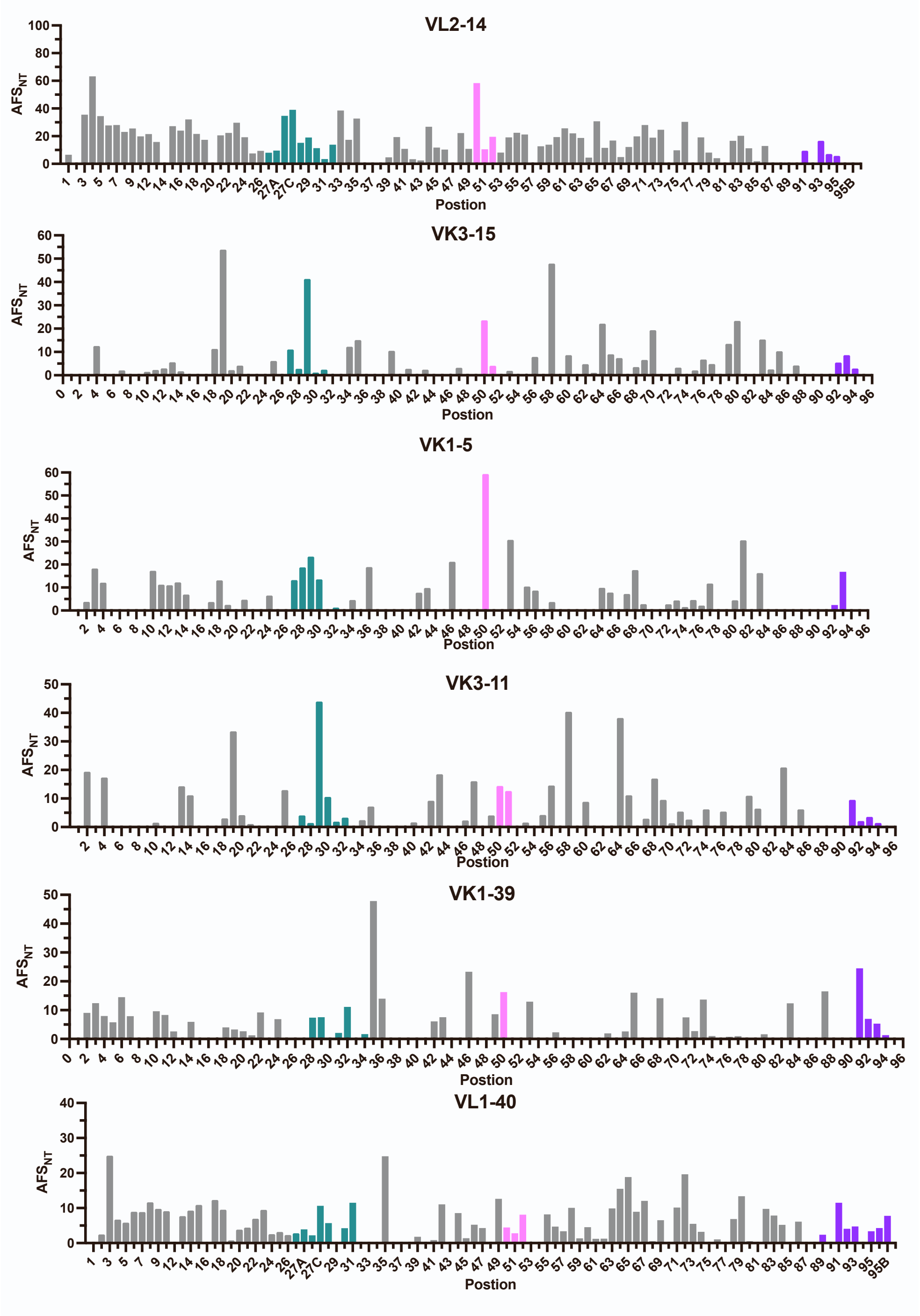

Figure S19. AFS$_{NT}$ for most common light chain germlines. AFS$_{NT}$ scores for the most common V-gene germlines in the OAS (IGLV2-14, IGKV3-15, IGKV1-5, IGKV3-11, IGKV1-39, IGLV1-40). All AFS$_{NT}$ scores were developed as described in Figure 5. They are S1-S2 for codons per position. The full V gene was searched with germline encoded CDRL1 and CDRL2 lengths, but length was allowed to vary for CDRL3 to capture more antibody sequences of interest.
